# Rapid evolution and molecular convergence in cryptorchidism-related genes associated with inherently undescended testes in mammals

**DOI:** 10.1186/s12862-021-01753-5

**Published:** 2021-02-10

**Authors:** Simin Chai, Ran Tian, Juanjuan Bi, Shixia Xu, Guang Yang, Wenhua Ren

**Affiliations:** School of Life Sciences, Nanjing Normal University, Nanjing, 210023 Jiangsu China

**Keywords:** Testicular descent, Cryptorchidism-related genes, Molecular convergence, Rapid evolution

## Abstract

**Background:**

The mammalian testis is an important male exocrine gland and spermatozoa-producing organ that usually lies in extra-abdominal scrotums to provide a cooler environment for spermatogenesis and sperm storage. Testicles sometimes fail to descend, leading to cryptorchidism. However, certain groups of mammals possess inherently ascrotal testes (i.e. testes that do not descend completely or at all) that have the same physiological functions as completely descended scrotal testes. Although several anatomical and hormonal factors involved in testicular descent have been studied, there is still a paucity of comprehensive research on the genetic mechanisms underlying the evolution of testicular descent in mammals and how mammals with ascrotal testes maintain their reproductive health.

**Results:**

We performed integrative phenotypic and comparative genomic analyses of 380 cryptorchidism-related genes and found that the mammalian ascrotal testes trait is derived from an ancestral scrotal state. Rapidly evolving genes in ascrotal mammals were enriched in the Hedgehog pathway—which regulates Leydig cell differentiation and testosterone secretion—and muscle development. Moreover, some cryptorchidism-related genes in ascrotal mammals had undergone positive selection and contained specific mutations and indels. Genes harboring convergent/parallel amino acid substitutions between ascrotal mammals were enriched in GTPase functions.

**Conclusions:**

Our results suggest that the scrotal testis is an ancestral state in mammals, and the ascrotal phenotype was derived multiple times in independent lineages. In addition, the adaptive evolution of genes involved in testicular descent and the development of the gubernaculum contributed to the evolution of ascrotal testes. Accurate DNA replication, the proper segregation of genetic material, and appropriate autophagy are the potential mechanisms for maintaining physiological normality during spermatogenesis in ascrotal mammals. Furthermore, the molecular convergence of GTPases is probably a mechanism in the ascrotal testes of different mammals. This study provides novel insights into the evolution of the testis and scrotum in mammals and contributes to a better understanding of the pathogenesis of cryptorchidism in humans.

## Background

The testis is a consequential male exocrine gland that produces spermatozoa and an endocrine gland that secretes sex hormones. For most mammals, both the testis and epididymis are located in the scrotum, which is outside of the body and protects the testis. The multi-version ‘cooling hypothesis’ [1, 2] suggests that the scrotum provides an environment 2–4 °C cooler than the normal body temperature; germinal epithelium and spermatozoa are acutely sensitive to heat [3], so this environment is optimal for spermatogenesis and sperm storage [4–6].

Some other hypotheses have been raised to explain the evolutionary origin of testicular descent and the function of the scrotum—e.g. the ‘training hypothesis’ argues that the scrotum exposes the sperm to a hostile environment to “train” it for further fertilization [7], Portmann [8] contends that the scrotum serves as a sexual signal in some mammals, and the ‘galloping hypothesis’ states that the scrotum originated in mammals that gallop or jump and protects spermatogenesis and sperm storage from consequent fluctuations in intra-abdominal pressure [9, 10].

Cryptorchidism (from Greek, meaning “hidden testicle”) is a failure of the testis to descend into the scrotal sac. For mammals with completely descended testes (CDT), cryptorchidism is a developmental defect that causes severe dysfunctions such as germ cell maldevelopment, asthenospermia, and imbalances in hormones such as testosterone (T) and anti-Müllerian hormone (AMH) [4]. Furthermore, cryptorchidism is associated with an increased risk of testicular malignancy and other diseases [5, 11]. Overall, cryptorchidism, a congenital malformation in most mammals, can affect normal male physiological functions.

Anatomical and physiological adaptations of the scrotum—e.g. thin skin, no subcutaneous fat, absence of hair/fur, and cremaster muscle—keep the epididymis and testis cool to maintain male fertility and health [12]. However, some mammals possess natural and properly functioning undescended testes (testes that do not descend completely or at all). For example, the platypus (*Ornithorhynchus anatinus*), a monotreme, has high intra-abdominal undescended testes (UDT) in the same initial position as the ovary in females and does not develop a scrotum [13]. Most species in Afrotheria—e.g. elephant (*Loxodonta africana*), cape golden mole (*Chrysochloris asiatica*), and manatee (*Trichechus manatus latirostris*)—have UDT [14–16], whereas the aardvark (*Orycteropus afer afer*) has incompletely descended testes (IDT) and lacks a scrotum [17]. In addition, armadillo (*Dasypus novemcinctus*; Xenarthra) and several lineages of Boreoeutheria (e.g. cetaceans, flying foxes, some pinnipeds, eulipotyphlans, and certain rodents) possess IDT [12].

In general, the CDT forms through a distinct and sequential two-phase descent [18], each phase of which involves multiple mechanical and hormonal factors. During the first phase, or the transabdominal phase, the testis is anchored to the inguinal region from a high abdominal position with the help of a swelling gubernaculum, the cranial suspensory ligament (CSL), T, and AMH [12, 19]. Early in the embryo development process, the gubernaculum primarily consists of a mesenchymal core and muscular outer layer, and has the potential to develop into a striated muscle bundle [20]. Later on, the gubernaculum can be found as a striated muscle bundle that eventually connects to the abdominal wall and scrotum [21, 22]. The gubernacular swelling reaction is mainly controlled by the insulin-like 3 protein (INSL3) and its receptor leucine-rich repeat-containing G protein coupled receptor 8 (RXFP2) [23].

During the second—or the inguino-scrotal—phase, the testis migrates into the scrotum. This process is regulated by a combination of gubernaculum, testosterone, calcitonin gene-related peptide (CGRP) released by the genitofemoral nerve (GFN), and intra-abdominal pressure [12, 24, 25]. A recent study associated the absence of testicular descent to the inactivation of *INSL3* and *RXFP2* in several UDT mammalian lineages like tenrec (*Echinops telfairi*), cape golden mole, cape elephant shrew (*Elephantulus edwardii*), and manatee [17]. However, the same study also showed that many other ascrotal mammals have full *INSL3* and *RXFP2* gene sequences, such as the elephant. This implies that the development and evolution of testicular descent in mammals likely involve polygene. Overall, both the etiology of cryptorchidism and the genetic mechanisms related to the evolution of testicular descent remain largely unknown.

This study analyzes cryptorchidism-related genes in mammals to investigate the mechanisms underlying developmental testicular descent and its adaptive importance in ascrotal mammals. This is important not only to understand the evolutionary mechanisms of testicular descent in mammals, but also to reveal the genetic mechanisms underlying cryptorchidism; thus, this study may be used to improve reproductive health in humans.

## Results

### Scrotal CDT is the ancestral state and was lost independently in many lineages

**Fig. 1:**
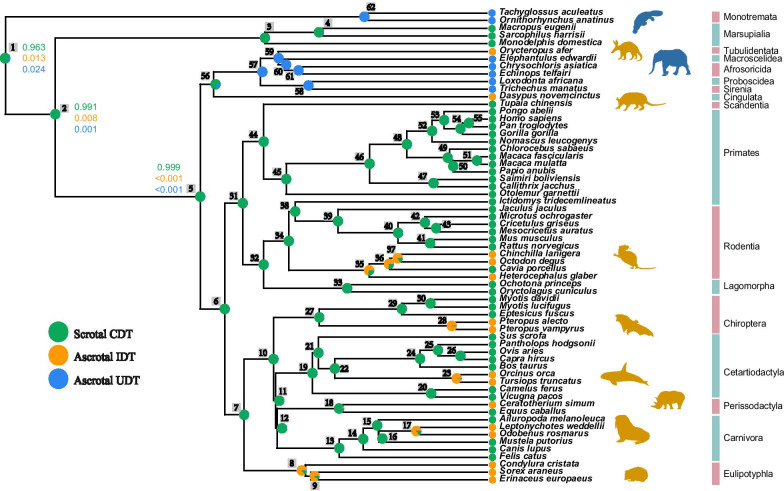
Evolution of testicular descent and the scrotum in mammals. Ancestral character states of the testis and scrotum were reconstructed using the ARD (All Rates Different) model. Each internal node in the phylogeny is numbered. UDT: undescended testis; IDT: incompletely descended testis; CDT: completely descended testis. The probability of each state at the ancestral nodes are shown in the pie charts. The value at each internal node is the calculated possibility of the state being scrotal CDT, ascrotal IDT, and ascrotal UDT. An order-level phylogeny is to the far right. All silhouettes are reproduced from PHYLOPIC (http://phylopic.org/)

We used a recent species tree [26] to map the phenotypic states of testicular position and the presence of a scrotum. The mapping shows that the testis and scrotum phenotypes are diverse across mammals (Fig. 1, Additional file 1: Table S1). A comparison of the evolutionary models found that ARD (All Rates Different), which treats all rates as different during trait evolution, was the best fitting model for testis and scrotum evolution in mammals (Additional file 1: Table S2). Reconstruction of the ancestral state suggested that scrotal CDT had a higher probability of appearing in deep ancestral nodes than did the undescended testis, e.g. the ancestor of mammals (node 1: 0.963), therians (node 2: 0.991), and placental mammals (node 5: 0.999) (Fig. 1). Moreover, the testis and scrotum phenotypes recovered several shifts from scrotal CDT to ascrotal UDT in internal nodes within Afrotheria, and to the ascrotal IDT in certain ancestral nodes of rodents, e.g. in the respective ancestors of cetaceans, megabats, and pinnipeds, plus the ancestral nodes within Eulipotyphla (Fig. 1).

### Molecular evolution of cryptorchidism-related genes involved in testicular descent

**Fig. 2:**
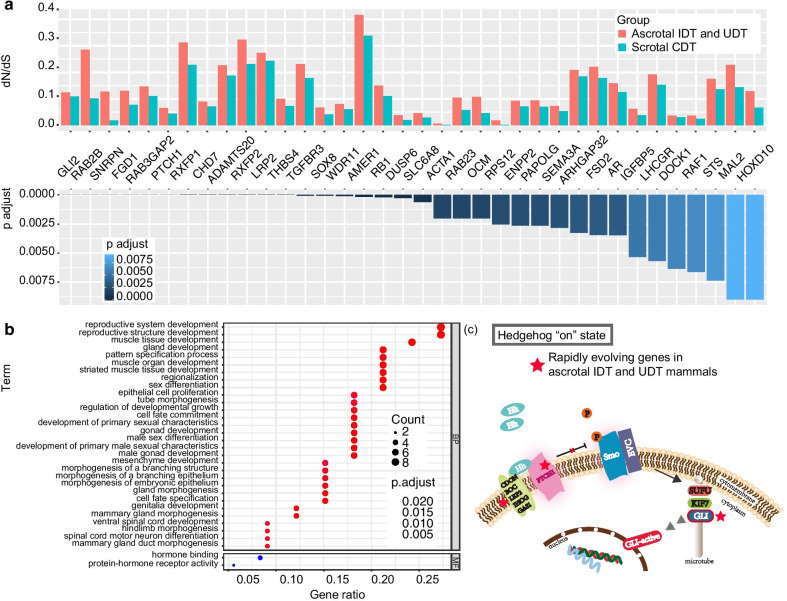
Rapidly evolving genes in ascrotal IDT and UDT mammals. **a** *dn/ds* values of 36 rapidly evolving genes in ascrotal species compared to scrotal testis mammals with adjusted p < 0.01 (corrected by FDR). **b** Gene Ontology enrichment of rapidly evolving genes in ascrotal mammals. Top 30 functional terms in biological process and all the terms in the molecular function class are shown. **c** Schematic description of Hedgehog signaling pathway when "Hedgehog" is on. Genes with a red star are rapidly evolving genes in IDT and UDT mammals

Branch model analysis in PAML detected 36 genes that evolved significantly (adjusted p < 0.01) increased molecular substitution rates in ascrotal IDT and UDT branches compared to CDT species (Fig. 2 and Additional file 1: Table S3). Among them, substitution rates in ascrotal mammals were up to 10.7 times greater than they were in scrotal ones. In addition, 33 out of these 36 genes were still rapidly evolved in ascrotal species in an expanded 62-mammal data set (Additional file 2: Fig S1), confirming the validity of the result (Additional file 1: Table S4). A further functional and pathway enrichment analysis revealed that 36 rapidly evolving genes were significantly enriched in the Hedgehog signaling pathway of Kyoto Encyclopedia of Genes and Genomes (KEGG) (Fig. 2 and Additional file 1: Table S5) and terms related to reproductive development (reproductive structure development, reproductive system development, male gonad development, development of primary male sexual characteristics, and male sex differentiation), muscle (muscle tissue development, striated muscle tissue development, and muscle organ development), and sex hormone receptors (protein-hormone receptor activity and hormone binding) of Gene Ontology (GO) (adjusted p < 0.05) (Fig. 2 and Additional file 1: Table S6).

Next, we detected genes with increased substitution rates in UDT mammals, which evolved as the most unique and extreme case of testis-scrotum phenotype. Forty-six genes had rapidly evolved in UDT species (Additional file 2: Fig S2 and Additional file 1: Table S7).

Phylogenetic generalized least squares (PGLS) regression was applied to detect any potential relationship between the evolutionary rates of cryptorchidism-related genes and testicular descent. Thirteen genes were found to be significantly associated: *MKX*, *TMEM74*, *BTBD1*, *SPPL2C*, *ATRX*, *NANOS1*, *AP3B2*, *IRF6*, *PRRG4*, *RAB3GAP2*, *DSCC1*, *EIF3A,* and *STS* (Table 1). Among them, chromatin-remodeling protein ATRX is involved in transcriptional regulation and telomere replication [27]. The gene *NANOS1* (Nanos C2HC-Type Zinc Finger 1), a member of the nanos family, is associated with spermatogenic impairment and translational regulation [28]. Further functional enrichment using GO annotations revealed terms related to sister chromatid cohesion (adjusted p < 0.05) (Additional file 1: Table S8 and Additional file 2: Fig S3).

**Table 1:** Significant association between evolutionary rates and testicular descent in PGLS

Gene	p value	r^2^	λ	AIC
*MKX*	0.01324	0.1071	1	36.04678
*TMEM74*	0.01632	0.09785	1	38.47006
*BTBD1*	0.0198	0.09318	1	36.79198
*SPPL2C*	0.0232	0.08585	1	39.11798
*ATRX*	0.0271	0.08224	1	37.36731
*NANOS1*	0.0278	0.08136	1	37.41327
*AP3B2*	0.03493	0.07189	1	39.86059
*IRF6*	0.03691	0.07	1	39.95986
*PRRG4*	0.04599	0.06253	1	40.35212
*RAB3GAP2*	0.04605	0.06382	1	38.32113
*DSCC1*	0.04713	0.0617	1	40.39561
*EIF3A*	0.04778	0.06255	1	38.38645
*STS*	0.04994	0.06101	1	38.46501

We used a branch-site model to test positively selected genes and amino acids in ascrotal IDT and UDT mammals. Evidence of positive selection was found in 13 ascrotal mammal genes (*FLNA*, *DOCK1*, *CSMD3*, *MCMBP*, *FANCE*, *FBXL18*, *ARMC4*, *DEPTOR*, *ACTA1*, *JAG1*, *AIMP2*, *RAF1*, and *CCDC73*) (Table 2). Enrichment analysis indicated that the functions of these 13 genes were significantly related to muscle (actin filament, sarcomere, contractile fiber part, myofibril, and contractile fiber), DNA replication (MCM complex), and GTPase (guanyl-nucleotide exchange factor complex) after multiple testing (adjusted p < 0.05) (Additional file 2: Fig S4 and Additional file 1: Table S9).

**Table 2:** Positively selected genes and sites in ascrotal IDT and UDT mammals detected by branch-site model

Gene	2∆ (lnL)	p value (< 0.05)	Adjusted p value (< 0.05)	ω value	Positively selected sites† (PP > 0.8)
*FLNA*	90.45473	< 0.0001	< 0.0001	19.77482	6 (0.902) 432 (0.859) 1115 (0.818) 1764 (0.876) 2058 (0.999) 2399 (0.912)
*DOCK1*	66.31111	< 0.0001	< 0.0001	3.01947	120 (0.948) 781 (0.899) 1817 (0.927)
*CSMD3*	45.53421	< 0.0001	< 0.0001	22.94946	391 (0.961) 2238 (0.874) 2314 (0.813) 3022 (0.934) 3105 (0.927) 3341 (0.853) 3416 (0.828) 3565 (0.990)
*MCMBP*	23.477574	< 0.0001	< 0.0001	29.13722	190 (0.871) 249 (0.856) 273 (0.986) 401 (0.867)
*FANCE*	21.201804	< 0.0001	< 0.0001	4.29612	59 (0.818) 150 (0.994) 192 (0.998) 265 (0.863) 427 (0.896)
*FBXL18*	21.03184	< 0.0001	< 0.0001	33.89202	19 (0.980) 160 (0.813)
*ARMC4*	18.634242	< 0.0001	0.0007	4.42802	194 (0.917) 386 (0.931) 667 (0.877) 978 (0.950)
*DEPTOR*	18.15214	< 0.0001	0.0009	4.59605	184 (0.908)
*ACTA1*	17.687794	< 0.0001	0.0010	3.40263	93 (0.818) 302 (0.962)
*JAG1*	15.491652	< 0.0001	0.0028	4.20226	1006 (0.853) 1136 (0.926)
*AIMP2*	15.104846	0.0001	0.0032	22.04281	
*RAF1*	13.280048	0.0003	0.0077	5.13164	
*CCDC73*	11.117498	0.0009	0.0229	3.61345	418 (0.946) 440 (0.901) 826 (0.998) 1027 (0.966)

^†^PPs of Bayes Empirical Bayes (BEB) analysis with P > 0.8 were regarded as amino acid candidates for selection

### UDT species-specific amino acid mutations and indels

**Fig. 3:**
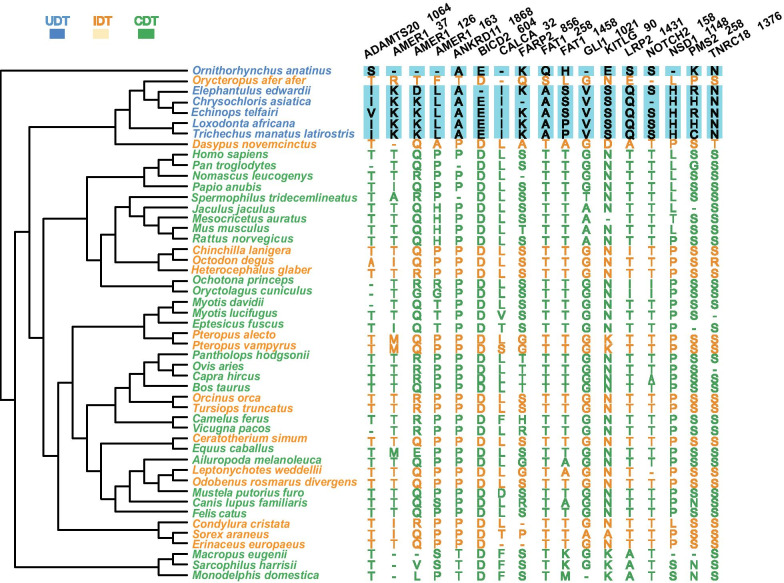
UDT mammal-specific amino acid mutations. Deduced and aligned amino acid sequences show unique mutations in UDT mammals compared to non-UDT species. Amino acid locations were deduced using the human gene as a reference. The corresponding sites in UDT mammals are illustrated with a blue background, IDT with beige, and CDT in green

**Fig. 4:**
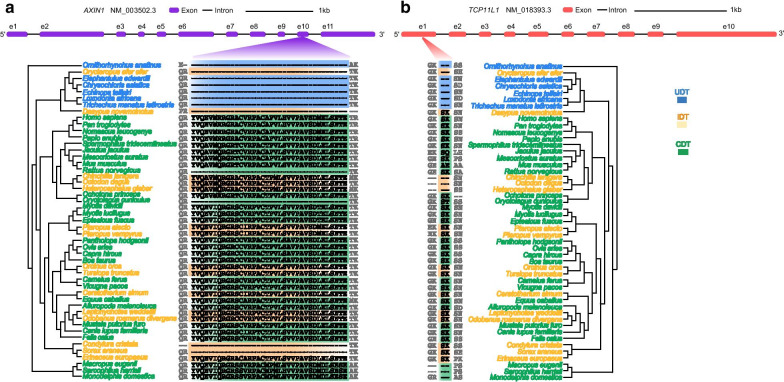
Indels in AXIN1 and TCP11L1. **a** A 108-bp deletion in exon 10 of *AXIN1* led to a loss of 36 amino acids in all UDT mammals plus four IDT species (*Orycteropus afer afer*, *Dasypus novemcinctus*, *Condylura cristata,* and *Sorex araneus*) and two CDT species (*Rattus norvegicus* and *Oryctolagus cuniculus*). **b** TCP11L1 has a six-bp-long deletion, leading to the loss of two amino acids in all UDT species. A two-amino-acid deletion also occurred in three IDT species (*Chinchilla lanigera*, *Octodon degus,* and *Heterocephalus glaber*) and marsupials in our dataset. UDT mammals are in blue, IDT in orange, and CDT in green

We failed to detect any specific mutations in ascrotal mammals (UDT or IDT species), but we did successfully identify 17 specific substitutions in 14 proteins of UDT mammals (Fig. 3). The protein AMER1 had three specific substitutions, FAT1 had two, and the other 12 proteins had one each. In addition, two indels were detected mainly in UDT mammals (Fig. 4): (1) a 108-bp deletion in *AXIN1* caused a 36-amino-acid fragment to be missing in all the UDT mammals and a small number of the IDT and CDT species and (2) a six-bp-long deletion in all UDT and some CDT species.

### Molecular convergence in ascrotal mammals

**Fig. 5:**
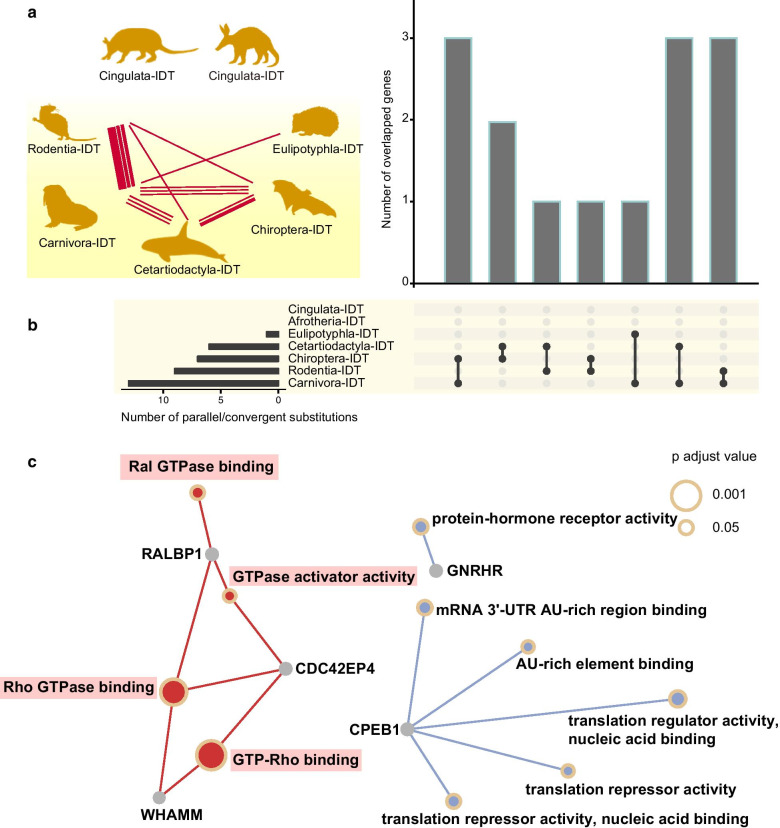
Convergent evolution in ascrotal IDT and UDT mammals. **a** The parallel/convergent amino acid substitutions among ascrotal IDT and UDT mammals. A protein harboring substitution(s) between two orders is denoted with a line. The boldness of the line indicates the number of substitutions that occurred. **b** Number of and overlapping proteins involving parallel/convergent substitution(s) among ascrotal species, visualized as an UpSet plot. **c** Functional enrichment of parallel/convergent substitution related genes. All silhouettes are reproduced from PHYLOPIC (http://phylopic.org/)

We initially observed convergent/parallel amino acid substitutions in 164 proteins in 176 IDT/UDT ascrotal branches (Additional file 1: Table S10 and Additional file 2: Fig S5). The Poisson test detected three parallel substitutions in the protein CPEB1 among different UDT branches, and 18 parallel changes were found in 13 proteins—AHSA2, FHL3, GNRHR, PROSER2, RALBP1, SERTAD2, WDPCP, C15orf40, CDC42EP4, CDT1, CPEB1, PGLS, and WHAMM—among the IDT branches (Additional file 1: Table S11). A functional enrichment analysis found that these 13 genes were related to small GTPase functions, such as GTP-Rho binding, Rho GTPase binding, and GTPase activator activity (Additional file 1: Table S12 and Fig. 5).

## Discussion

Most mammals have completely descended testes (CDT). Although the failure of testicular descent leads to cryptorchidism, certain groups of mammals possess incompletely descended (IDT) or undescended testes (UDT) that function normally. The evolution of the scrotum and testicular descent in mammals has long been the subject of scientific interest in a wide range of fields, including medicine, developmental biology, and evolutionary biology. However, little is known about this topic from an evolutionary perspective. Here, comparative genomics and evolutionary analyses of cryptorchidism-related genes provide some novel evidence for the evolutionary trajectory of testicular descent and potential mechanisms driving normal physiological functions for IDT and UDT in mammals.

### The ascrotal testis evolved multiple times independently in mammals

It has been suggested that descended testes and the scrotum provide an optimal cooling environment for spermatogenesis and sperm storage, since they are cooler in temperature than the core body [1, 2, 29]. However, propitious testicular descent into the scrotum could be a sophisticated process that is physiologically, developmentally, and evolutionarily costly [30]. Mammals evolved divergent scrotal CDT as well as ascrotal IDT and UDT. However, unlike in hard tissues, it is difficult to trace the evolutionary history of these organs because they do not fossilize. Nevertheless, inferring ancestral character states by mapping various phenotypes of living taxa onto a phylogeny could suggest the evolutionary trajectory of a certain trait [31].

Our ancestral state reconstruction showed that several deep ancestors of mammals possessed scrotal CDT (Fig. 1). The derived characters of ascrotal IDT and UDT were inferred to occur in multiple lineages—e.g. Monotremata, Afrotheria, Cingulata, Rodentia, Chiroptera, Cetartiodactyla, Pinnipedia, and Eulipotyphla. Although Kleisner et al. [15] and Lovegrove [16] suggested that the scrotal testis is a derived state in mammals, Werdelin and Nilsonne [14] and Sharma et al. [17] argue that the scrotal CDT is the ancestral state and was subsequently lost in separate lineages. Overall, our reconstruction of the evolutionary history of testicular descent and the presence of the scrotum relied on a high order-level species coverage and well-accepted phylogeny, providing more credible information about the plesiomorphic scrotal testis in mammals. Moreover, the finding that the ascrotal testis evolved independently in mammals is a fascinating instance of convergent evolution.

### Genetic mechanisms driving the evolution of testicular descent in mammals

The two phases of testicular descent are regulated by a combination of mechanical and hormonal factors [18]. Dysplasia of the gubernacula, a pair of structures needed to steer the descent, yielded abnormal testicular descent. Previous studies have also found that mesenchymal, fibroblast, and muscle cells are involved in gubernaculum development [21, 22].

In the present study, we identified a series of rapidly evolving genes in ascrotal IDT and UDT mammals that were enriched in functions related to muscle and striated muscle development (Fig. 2, Additional file 1: Table S3, and Table S6). Positively selected genes in the group of ascrotal species were also overrepresented in GO terms related to muscular components (Additional file 1: Table S9 and Additional file 2: Fig S4). These findings imply that evolutionary changes in genes related to the muscular gubernaculum were involved in testicular descent. This is similar to the finding from Barthold et al. [32] that the differentially expressed genes of the gubernaculum in wild-type and cryptorchid rat fetuses were enriched in categories related to muscle development. Combined, this evidence suggests that muscle-related genes played some important role in the evolution of testicular descent in mammals, specifically by contributing to mechanical traction.

The process of testicular descent also involves a series of hormones. T, which is produced by Leydig cells, could induce CSL regression and cause the testis to descend into the first phase. More importantly, during the second phase, androgens (including T) are the predominant hormonal controllers [12]. Moreover, T plays a large role in a paracrine manner to stabilize the Wolffian ducts and further systemically masculinize the external genitalia [33].

Desert hedgehog (Dhh) (Hedgehog protein family) has been reported to play an important role in regulating T secretion and testis development. Dhh is produced by Sertoli cells and can regulate the proliferation and differentiation of Leydig cells and functions of T secretion [34, 35]. A missense mutation in the rat Hedgehog pathway resulted in androgen deficiency and a decrease in the number of Leydig cells with impaired functions [36]. Our finding that the rapidly evolving genes in ascrotal mammals were overrepresented in the Hedgehog signaling pathway (Fig. 2, Additional file 1: Table S3, and Table S5) aligns with the critical role of the Hedgehog signaling pathway in T secretion and testis development.

### Possible mechanisms for maintaining health in IDT and UDT mammals

The testis is a crucial producer of spermatozoa and sex hormones in males. However, cryptorchidism caused by testicular maldescent is a heterogeneous disorder associated with not only the macroscopic abnormal location of testes, but also further postnatal abnormalities. For example, cryptorchidism is one of the most common causes of infertility in men; it is characterized by depression of spermatogenesis [37] and reduction in the number of germ cells [38]. Abnormal autophagy [39] and DNA damage [40] were suggested to be involved in the impairment of spermatozoa in the cryptorchid testis. The present study uncovered several lines of evidence for strategies by which ascrotal IDT and UDT mammals keep normal reproductive capacity under “cryptorchid conditions.”

First, genes involved in spermatogenesis were associated with healthy UDT. G1021V in the protein GLI1 and E/S90N in KITLG were identified as UDT mammal-specific amino acid mutations. The zinc finger transcription factor GLI1 is a member of the GLI family, whose overexpression or mutation causes disease in humans and mice [41, 42]. There is evidence that GLI1 mediates Desert hedgehog (Dhh) signaling in male mouse testes during spermatogenesis [43]. The KIT/KITLG signaling system is essential for the proliferation, meiosis, migration, survival, and maturation of germ cells in testes [44, 45]. It has been reported that polymorphisms in KITLG are likely associated with germ cell tumors [46]. Although residue 1021 of GLI1 is not located in a putative functional region and nonsynonymous mutation of residue 90 is an unreported polymorphism case, both might be targeted in response to spermatogenesis via unknown cis-regulations.

We also found that the genes *ATRX* and *NANOS1*, which function in spermatogenesis in the testis, evolved an association with testicular descent. *ATRX* (on the X-chromosome, responsible for alpha-thalassaemia and mental retardation) is believed to play a role in testicular development, since the majority of mutations in ATRX result in genital abnormalities [47]. Tang et al. [48] suggested that ATRX might also contribute to adult spermatogenesis in human. Mutations in the gene *NANOS1* are associated with spermatogenic failure and oligoasthenoteratozoospermia [49].

Of the genes with UDT mammal-specific indels, T-complex 11 like 1 (*TCP11L1*) is involved in cryptorchidism. Seabra et al. [50] described a cryptorchidism patient with azoospermia presenting a microdeletion at 11p13 in *TCP11L1*. Liu et al. [51] found that TCP11L1 shares its functional domain with and has similar subcellular localization to the TCP11 protein, suggesting that the two proteins have a similar role in spermatogenesis. Several studies have demonstrated that, similar to mutations and insertions, deletions in a protein region might enhance that protein’s molecular function if the region effects it; this also occurs with *cis-* or *trans-*acting regulation [52, 53]. It is likely that this unique deletion of TCP11L1 in UDT species enhances spermatogenic capability, but further functional assays are needed to confirm this.

Second, genes contributing to DNA repair and genome stability maintenance were found to be involved in potential mechanisms maintaining health in UDT mammals. Germ cells pass genetic information onto descendants, and this requires that replication is accurate and their genome is perfectly stable [54]. Obstacles to replication might cause genomic instability and cancer formation [55]. However, the physiological environment provided by the undescended testis challenges the enzymes and cellular mechanisms that appear to be well adapted to the lower temperature in the CDT [56]. We found that PMS2 possesses a UDT species-specific mutation at position 258. A previous study found that PMS2 plays a crucial role not only in the post-replicative DNA mismatch repair system, but also in a process that induces cell cycle arrest and could lead to apoptosis in the case of major DNA damages—this is known as DNA damage signaling [57]. Along with DNA replication repair, sister chromatid cohesion in the meiotic process is another crucial mechanism for maintaining genome stability [58]. Our results showed that genes that evolved in association with testicular descent were significantly enriched in functions that maintain sister chromatid cohesion (Additional file 1: Table S8 and Additional file 2: Fig S3), a biological process in which sister chromatids of a replicated chromosome become tethered to each other. Cohesion in eukaryotic cells appears to lie at the heart of the meiotic process because it is compulsory for the repair of recombinogenic lesions and for chromosome segregation in dividing cells during meiotic anaphase [59, 60].

Third, genes involved in autophagy might help maintain health in UDT mammals. Well-adjusted germ cell proliferation and death are highly ordered in testicular spermatogenesis [61, 62]. However, testicular heating suppresses spermatogenesis and leads to increasing germ cell degeneration and death [63, 64]. Autophagy involves a biological process of self-cannibalization via lysosomal degradation, namely nonapoptotic cellular demise [65]. Zheng et al. [66] found that spermatogenesis was impaired in a surgery-induced cryptorchid mouse model; they also found cryptorchidism-induced autophagy and apoptosis synchronously promoting germ cell death. Notably, there have hardly been any reports of over-autophagy in naturally ascrotal mammals. Therefore, we believe that, to maintain homeostasis in testes and germ cells, ascrotal species might adjust their autophagy levels to adapt to the high ambient temperature of the undescended testes. This is supported by the significant correlation of autophagy associated genes *TMEM74* and *RAB3GAP2* in the PGLS analysis and a rapidly evolving gene *RAB7A* in UDT mammals (Table 1 and Fig. 2). Transmembrane protein 74 (*TMEM74*) might be an important element promoting autophagy under cell stress conditions, because the knockdown of this gene hampers the cell’s autophagy function when starvation is imposed [67]. RAB3GAP2 and RAB7A are encoded by family members of RAB, which is a group of GTP-binding proteins that regulate vesicular transport. Both genes are involved in the autophagy pathway [68, 69].

### Molecular convergence of the ascrotal IDT and UDT in GTPase

For decades, researches have demonstrated that phenotypic convergence can result from convergent molecular mechanisms [70]. In our study, phylogenetic reconstruction revealed that the ascrotal testis evolved in different mammalian lineages independently, and that this could be regarded as convergent evolution. Genes harboring convergent/parallel substitutions in the ascrotal mammals were found to be significantly enriched in small GTPase-related terms (Additional file 1: Table S12 and Fig. 5). Small GTPases are a large family of molecular switches that play a pivotal role in various cellular processes [71]. Specifically, recent advances have suggested that Ras GTPases are involved in testicular descent [72]. Syndromes such as Noonan, Cardiofaciocutaneous, LEOPARD, and Costello, which are characterized as cryptorchidism, are RASopathies [72]. Moreover, GTPase-mediated signal transduction was identified in the functional analysis of the differentially expressed genes of wild-type and cryptorchid rats [32]. A previous study argues that Rho GTPases are critical for cytoskeletal reorganization and myogenesis [73]. This is consistent with the roles of *INSL3/RXFP2* and other candidate genes in regulating myogenesis and muscle development in the development of gubernaculum during testicular descent [32]. Hence, it could be hypothesized that small GTPases drive the convergent evolution of the ascrotal testis in different mammals, playing an important role in the development of gubernaculum via myogenesis.

Nevertheless, the development of testicular descent is a complicated process involving many genes and pathways. Future studies using genome-wide scans are needed to elucidate the molecules and mechanisms dictating the evolution of testicular descent in mammals and test the candidate genes by functional analyses. Additionally, primates, which have a relatively low prevalence in the evolutionary history of the Mammalia [74], were overrepresented in this analysis. A more precise and comprehensive sampling that is proportional to the distribution of species in the Mammalia class is needed to remedy any potential ascertainment bias in the present study.

## Conclusions

Our study combined phenotypic evolution and comparative genomics investigations of 380 cryptorchidism-related genes in mammals and found that the scrotal testis is the ancestral state in mammals and the ascrotal testis evolved multiple times independently. More importantly, the rapidly evolving and positively selected genes that we found suggest that the derived status of the ascrotal IDT and UDT phenotypes in mammals can be attributed to the adaptive evolution of genes involved in testicular descent and muscle development. Moreover, we demonstrated that accurate DNA replication, high genome stability, and appropriate autophagy are likely the mechanisms by which ascrotal mammals maintain normal spermatogenesis and physiological health. Our results suggest that small GTPases are associated with molecular mechanisms that contribute to the convergent phenotype of ascrotal testes in different mammals. Our study provides some novel insights into the evolution of testicular descent in mammals, contributes to a better understanding of the pathogenesis of cryptorchidism in humans, and offers further experimental validation for these candidate genes and residues.

## Methods

### Data collection

Data on the presence and testicular position of the scrotums of 62 representative mammals in the UCSC 100-way multiple alignments (http://hgdownload.soe.ucsc.edu/downloads.html) and the monotreme echidna (*Tachyglossus aculeatus*) were taken from publications (Additional file 1: Table S1). Three categories of testicular position were classified: completely descended testis (CDT), incompletely descended testis (IDT), and undescended testis (UDT) (Fig. 1 and Additional file 1: Table S1). 380 cryptorchidism-related protein coding genes screened from *The Cryptorchidism Gene Database version 3* [75] were collected. Homologous exon alignments of these cryptorchidism-related genes were gathered from UCSC human 100-way multiple alignments at http://genome.ucsc.edu/. The entire transcripts were concatenated by exon-employing custom perl scripts. The longest transcript was retained for genes with multiple splice variants. To improve the quality of sequence alignments and subsequent evolutionary analyses, the nucleotide alignments were realigned by PRANK v.170427 in codon mode [76]. Sequences were then treated with Gblocks [77]. Incomplete codons and premature stop codons were prohibited. Based on the criteria that (1) at least one representative species for each main mammalian order was chosen, (2) all the focused “cryptorchidism” (IDT + UDT) species were included, and (3) species with higher quality genomes were kept, several CDT species which were mainly from the overrepresented primates were removed. Sequences from 49 mammals (28 CDT species, 15 IDT, and 6 UDT species) covering 16 orders (Primates, Rodentia, Lagomorpha, Cetartiodactyla, Perissodactyla, Carnivora, Chiroptera, Eulipotyphla, Proboscidea, Sirenia, Afrosoricida, Macroscelidea, Tubulidentata, Cingulata, Marsupialia, and Monotremata) (Additional file 1: Table S1, Additional file 2: Fig S5) were used in the evolutionary analyses described below.

### Ancestral state reconstruction

The discrete morphologies of the scrotum and testis used in this study for extant taxa were assigned according to previous studies (Additional file 1: Table S1). Ancestral character reconstructions of mammalian scrotum presence and testis position were performed via the phytools and ape packages in R statistical software [78, 79]. We first tested the fitness of the following models: Equal Rate model (ER): discrete character evolution in which a single parameter governs all transition rates; All Rates Different model (ARD): all possible transitions can occur at different rates; and Symmetrical Rates model (SYM): forward and reverse transitions share the same parameter. We then used the model with the best AIC score to reconstruct the ancestral state of mammalian scrotum presence and testis position. We used a well-supported mammal phylogeny from TimeTree (http://www.timetree.org/) [26] as the input tree.

### Selective pressure test

Rapidly evolved and positively selected genes generally contribute to adaptive evolution in response to new and changed environments [80]. To test whether heterogeneity in the evolutionary rates of cryptorchidism-related genes among mammals are correlated with different testis positions, we undertook a suite of analyses to detect differences in selection intensity and positive selection using codeml in the PAML package [81].

A false discovery rate (FDR) correction was applied to conservatively account for multiple testing. First, the branch model was recruited to examine ω (the ratio of nonsynonymous to synonymous substitution rates) values between each of two groups of branches. A nested model comparison between ascrotal and scrotal testis mammals—including a two-ratio model (in which scrotal and ascrotal mammals have different ω values) and a one-ratio model (in which all mammals have one ω value)—was used to evaluate rapidly evolving genes in ascrotal IDT and UDT mammals. Another branch-specific comparison was made between IDT and UDT species within ascrotal mammals.

Second, the branch-site model was used to detect positively selected genes and amino acids across the combined IDT and UDT branches. The ω value was used to describe selection pressure: ω < 1, ω = 1, and ω > 1 indicated purified selection, neutral evolution, and positive selection, respectively.

### Association analysis between sequence evolution and phenotype

To investigate the potential relationship between gene evolutionary rates and testicular and scrotal evolution in mammals, we employed a PGLS regression in the caper package of R [82]. We used the root-to-tip ω value to access the evolutionary rate of each coding sequence because it contains more evolutionary history than does the terminal-branch ω value [83]. The free ratio model of the codeml program in PAML was used to estimate the ω values of internal and terminal branches. We used a binary state combination of the scrotal CDT and ascrotal IDT and UDT, with relevant characters shown in Additional file 1: Table S1. The lambda (λ) value, used as a quantitative measure of phylogenetic signals, was estimated by the maximum likelihood method [84].

### Shared specific amino acids and convergent/parallel substitutions in UDT species

Species-specific amino acid mutations and peptide fragment indel (insertion and deletion) events have been inferred to be associated with functional, physiological, and phenotypic changes [85, 86]. For each amino acid site within a protein, the shared UDT species-specific mutations refer to the amino acids along all UDT branches that are different from other species. The shared specific substitutions were identified by strict identity. To identify shared specific amino acid mutations in the UDT species, in-house perl scripts were employed on each column of the trimmed amino acid alignments.

It has been suggested that convergent phenotypes can be mediated by parallel and convergent amino acid substitutions [87–89]. Convergent substitution occurs when different amino acids at a specific amino acid site from two distantly-related branches converge, whereas parallel substitution occurs when the same amino acid in two branches are derived from another same amino acid in independent ancestors [90]. Convergent/parallel amino acid substitutions in ascrotal branches were detected via the method described in Zou and Zhang [90].

For each gene, the ancestral node sequence was first reconstructed using the codeml program in the PAML package [81]. Second, a tree with branch lengths was extracted from the output file from the abovementioned calculation. Then, the relative evolutionary rates of all amino acid sites within one gene were calculated using the aaml model in codeml. The frequency of each amino acid at each site was counted for each gene. Using this method [90], all observed and expected cases of convergent and parallel amino acid substitutions were calculated under the recommended *JTT-f*_*gene*_ matrix. We focused on and filtered the convergent/parallel substitutions between each of two ascrotal branches. Finally, a Poisson test was employed to identify the significance between the observed and expected numbers of substitutions in ascrotal branches.

### Enrichment analysis

We used the over-representation tests enrichGO and enrichKEGG incorporated into clusterProfiler version 3.6.0 [91] to analyze Gene Ontology (GO) and KEGG enrichment, respectively. Benjamini and Hochberg (BH) multiple test correction [92] was performed to adjust p values and decrease the likelihood of false positives.

## Supplementary Information

**Additional file 1: Table S1.** The presence of scrotum and the testicular position of 63 representative mammals. **Table S2.** Model comparison of the evolution of testicular descent in the ancestral state reconstruction. **Table S3.** Rapidly evolved genes in ascrotal testis mammals. **Table S4.** Rapidly evolving genes in a 62-species mammalian data set. **Table S5.** KEGG enrichment of rapidly evolving genes in ascrotal mammals. **Table S6.** GO enrichment of rapidly evolving genes in ascrotal mammals. **Table S7.** Rapidly evolving genes in UDT mammals. **Table S8.** GO enrichment of genes evolved with significant regression with testicular descent. **Table S9.** GO enrichment of positively selected genes in ascrotal IDT and UDT mammals. **Table S10.** The observed convergent/parallel amino acid substitutions in the ascrotal IDT and UDT branches. **Table S11.** Parallel and convergent amino acid substitutions in ascrotal UDT and IDT mammals. **Table S12.** GO enrichment of genes exhibit parallel/convergent substitutions in IDT mammals.

**Additional file 2: Fig S1.** The expanded 62-mammal data set. **Fig S2.** Rapidly evolving genes in UDT mammals. **Fig S3.** Functional enrichment of genes that evolved significantly correlated to character of testis position (p value < 0.05). **Fig S4.** Top 10 GO function enrichment of positive selected genes in ascrotal mammals. **Fig S5.** The working tree with numbered internal nodes for identifying convergent/parallel amino acid substitutions.

## Data Availability

All data generated or analyzed during this study are included in this published article and its supplementary information files.

## Abbreviations

T: Testosterone
AMH: Anti-Müllerian hormone
CDT: Completely descended testis
UDT: Undescended testis
IDT: Incompletely descended testis
CGRP: Calcitonin gene-related peptide
GFN: Genitofemoral nerve
ER: Equal rate model
ARD: All rates different model
SYM: Symmetrical rates model
UCSC: University of California, Santa Cruz
FDR: False discovery rate
PGLS: Phylogenetic generalized least squares
GO: Gene Ontology
KEGG: Kyoto Encyclopedia of Genes and Genomes
BH: Benjamini and Hochberg
CSL: Cranial suspensory ligament
Dhh: Desert hedgehog
