# Supplementary material for: Rapid evolution and molecular convergence in cryptorchidism-related genes associated with inherently undescended testes in mammals
Source: BMC Ecol Evol. 2021 Feb 10;21:22. doi: 10.1186/s12862-021-01753-5 (PMC7877101; doi:10.1186/s12862-021-01753-5)
Supplement: Supplementary file 1 — Additional file 1: Table S1. The presence of scrotum and the testicular position of 63 representative mammals. Table S2. Model comparison of the evolution of testicular descent in the ancestral state reconstruction. Table S3. Rapidly evolved genes in ascrotal testis mammals. Table S4. Rapidly evolving genes in a 62-species mammalian data set. Table S5. KEGG enrichment of rapidly evolving genes in ascrotal mammals. Table S6. GO enrichment of rapidly evolving genes in ascrotal mammals. Table S7. Rapidly evolving genes in UDT mammals. Table S8. GO enrichment of genes evolved with significant regression with testicular descent. Table S9. GO enrichment of positively selected genes in ascrotal IDT and UDT mammals. Table S10. The observed convergent/parallel amino acid substitutions in the ascrotal IDT and UDT branches. Table S11. Parallel and convergent amino acid substitutions in ascrotal UDT and IDT mammals. Table S12. GO enrichment of genes exhibit parallel/convergent substitutions in IDT mammals. [file 12862_2021_1753_MOESM1_ESM.pdf]

Supplementary tables

Table S1 The presence of scrotum and the testicular position of 63 representative mammals.

Order	Common name	Scientific name	abbrev	Presence of scrotum and testicular position*	Literatures†
Primates	Human	★*Homo sapiens*	hg38	Scrotal CDT	a, b, c
	Chimp	★*Pan troglodytes*	panTro4	Scrotal CDT	b, c
	Gorilla	*Gorilla gorilla gorilla*	gorGor3	Scrotal CDT	b, c
	Orangutan	*Pongo pygmaeus abelii*	ponAbe2	Scrotal CDT	b, c
	Gibbon	★*Nomascus leucogenys*	nomLeu3	Scrotal CDT	b, c
	Rhesus	*Macaca mulatta*	rheMac3	Scrotal CDT	a, b
	Crab eating macaque	*Macaca fascicularis*	macFas5	Scrotal CDT	b
	Baboon	★*Papio anubis*	papAnu2	Scrotal CDT	b
	Green monkey	*Chlorocebus sabaeus*	chlSab2	Scrotal CDT	b
	Marmoset	*Callithrix jacchus*	calJac3	Scrotal CDT	b
	Squirrel monkey	*Saimiri boliviensis*	saiBol1	Scrotal CDT	b
	Bushbaby	*Otolemur garnettii*	otoGar3	Scrotal CDT	b
Scandentia	Chinese tree shrew	*Tupaia chinensis*	tupChi1	Scrotal CDT	c, d, e
Rodentia	Squirrel	★*Spermophilus tridecemlineatus*	speTri2	Scrotal CDT	b
	Lesser Egyptian jerboa	★*Jaculus jaculus*	jacJac1	Scrotal CDT	b
	Prairie vole	*Microtus ochrogaster*	micOch1	Scrotal CDT	b
	Chinese hamster	*Cricetulus griseus*	criGri1	Scrotal CDT	b
	Golden hamster	★*Mesocricetus auratus*	mesAur1	Scrotal CDT	b
	Mouse	★*Mus musculus*	mm10	Scrotal CDT	a, b
	Rat	★*Rattus norvegicus*	rn6	Scrotal CDT	a, b

	Naked mole rat	★*Heterocephalus glaber*	hetGla2	Ascrotal IDT	b
	Cavia porcellus	*Cavia porcellus*	cavPor3	Scrotal CDT	c
	Chinchilla	★*Chinchilla lanigera*	chiLan1	Ascrotal IDT	a, b
	Brush tailed rat	★*Octodon degus*	octDeg1	Ascrotal IDT	b
Lagomorpha	Rabbit	★*Oryctolagus cuniculus*	oryCun2	Scrotal CDT	b, d
	Pika	★*Ochotona princeps*	ochPri3	Scrotal CDT	b, d
Cetartiodactyla	Pig	*Sus scrofa*	susScr3	Scrotal CDT	b, d
	Alpaca	★*Vicugna pacos*	vicPac2	Scrotal CDT	b, d
	Wild bactrian camel	★*Camelus ferus*	camFer1	Scrotal CDT	b, d
	Dolphin	★*Tursiops truncatus*	turTru2	Ascrotal IDT	a, b
	Killer whale	★*Orcinus orca*	orcOrc1	Ascrotal IDT	a, b
	Tibetan antelope	★*Pantholops hodgsonii*	panHod1	Scrotal CDT	b, d
	Cow	★*Bos taurus*	bosTau8	Scrotal CDT	b, d
	Sheep	★*Ovis aries*	oviAri3	Scrotal CDT	b, d
	Domestic goat	★*Capra hircus*	capHir1	Scrotal CDT	b, d
Perissodactyla	Horse	★*Equus caballus*	equCab2	Scrotal CDT	b, d
	White rhinoceros	★*Ceratotherium simum*	cerSim1	Ascrotal IDT	b, d
Carnivora	Cat	★*Felis catus*	felCat8	Scrotal CDT	b, c, d
	Dog	★*Canis lupus familiaris*	canFam3	Scrotal CDT	b, c, d
	Ferret	★*Mustela putorius furo*	musFur1	Scrotal CDT	b, c, d
	Panda	★*Ailuropoda melanoleuca*	ailMel1	Scrotal CDT	b, d
	Pacific walrus	★*Odobenus rosmarus divergens*	odoRosDiv1	Ascrotal IDT	b, d
	Weddell seal	★*Leptonychotes weddellii*	lepWed1	Ascrotal IDT	b, d
Chiroptera	Black flying fox	★*Pteropus alecto*	pteAle1	Ascrotal IDT	b, d
	Megabat	★*Pteropus vampyrus*	pteVam1	Ascrotal IDT	b, d
	Big brown bat	★*Eptesicus fuscus*	eptFus1	Scrotal CDT	b, d
	David's myotis	★*Myotis davidii*	myoDav1	Scrotal CDT	b, d

	Microbat	✭*Myotis lucifugus*	myoLuc2	Scrotal CDT	b, d
Eulipotyphla	Hedgehog	✭*Erinaceus europaeus*	eriEur2	Ascrotal IDT	a, b
	Shrew	✭*Sorex araneus*	sorAra2	Ascrotal IDT	a, b
	Star-nosed mole	✭*Condylura cristata*	conCri1	Ascrotal IDT	a, b
Proboscidea	Elephant	✭*Loxodonta africana*	loxAfr3	Ascrotal UDT	a, b, d
Sirenia	Manatee	✭*Trichechus manatus latirostris*	triMan1	Ascrotal UDT	a, b, d
Afrosoricida	Cape golden mole	✭*Chrysochloris asiatica*	chrAsi1	Ascrotal UDT	a, b, d
	Tenrec	✭*Echinops telfairi*	echTel2	Ascrotal UDT	a, b, d
Macroscelidea	Cape elephant shrew	✭*Elephantulus edwardii*	eleEdw1	Ascrotal UDT	a, b, d
Tubulidentata	Aardvark	✭*Orycteropus afer afer*	oryAfe1	Ascrotal IDT	a, b, d, f
Cingulata	Armadillo	✭*Dasypus novemcinctus*	dasNov3	Ascrotal IDT	a
Marsupialia	Opossum	✭*Monodelphis domestica*	monDom5	Scrotal CDT	a, b, c
	Tasmanian devil	✭*Sarcophilus harrisii*	sarHar1	Scrotal CDT	a, b
	Wallaby	✭*Macropus eugenii*	macEug2	Scrotal CDT	a, b
Monotremata	Platypus	✭*Ornithorhynchus anatinus*	ornAna1	Ascrotal UDT	a, b
	Echidnas	✭*Tachyglossus aculeatus*	tacAcu	Ascrotal UDT	g, h

✭ Species for evolutionary analyses.

∗ Abbreviation of the position of testis: completely descended testis (CDT), incompletely descended testis (IDT), undescended testis (UDT).

† Code for literatures:

a. Hutson J M, Thorup J M, Beasley S W. Descent of the Testis. Springer, 2015.
b. Lovegrove B G. Cool sperm: why some placental mammals have a scrotum. Journal of evolutionary biology, 2014, 27(5): 801-814.
c. Williams M P L, Hutson J M. The phylogeny of testicular descent. Pediatric surgery international, 1991, 6(3): 162-166.
d. Kleisner K, Ivell R, Flegr J. The evolutionary history of testicular externalization and the origin of the scrotum. Journal of biosciences, 2010, 35(1): 27-37.
e. Peng Y Z, et al. Biology of Chinese Tree Shrews (*Tupaia belangeri chinensis*). Yunnan Science and Technology Press, 1991.
f. Foresta C, Zuccarello D, Garolla A, et al. Role of hormones, genes, and environment in human cryptorchidism. Endocrine reviews, 2008, 29(5): 560-580.

g. Griffiths, M. The biology of the monotremes. Elsevier, 2012.
h. Temple-Smith P, Grant T. Uncertain breeding: a short history of reproduction in monotremes. Reproduction, Fertility and Development, 2001, 13(8), 487-497.

Table S2. Model comparison of the evolution of the testicular descent in the ancestral state reconstruction.

Model	AIC
ER (equal-rates model of where a single parameter governs all transition rates)	80.373747
ARD (all-rates-different model where each rate is a unique parameter)	78.914483
SYM (symmetrical model where forward and reverse transitions share the same parameter)	79.519909

Table S3 Rapidly evolved genes in ascrotal IDT and UDT mammals.

EntrezID	Gene	lnL (one ratio)	lnL (two ratio)	*ω* in scrotal CDT mammals	*ω* in ascrotal IDT and UDT mammals	p value	p adjust (<0.01)
2736	*GLI2*	-2990.12292	-66405.91758	0.10902	0.12394	0	0
84932	*RAB2B*	-6408.340642	-6379.476557	0.10156	0.28532	3.0087E-14	3.56183E-12
6638	*SNRPN*	-5263.892745	-5237.31576	0.0181	0.12779	3.0842E-13	2.7335E-11
2245	*FGD1*	-26474.56541	-26452.8082	0.07763	0.12941	4.20844E-11	2.49092E-09
25782	*RAB3GAP2*	-53496.342	-53478.85709	0.10985	0.14689	3.34857E-09	1.32139E-07
5727	*PTCH1*	-42643.64176	-42626.55777	0.04411	0.06513	5.05538E-09	1.7963E-07
59350	*RXFP1*	-30774.97898	-30761.41637	0.22779	0.31284	1.90693E-07	5.57077E-06
55636	*CHD7*	-83782.5773	-83769.0813	0.07077	0.08958	2.04299E-07	5.57077E-06
80070	*ADAMTS20*	-77115.93255	-77102.73027	0.18666	0.22647	2.76886E-07	7.02393E-06
122042	*RXFP2*	-27069.3129	-27056.50092	0.23031	0.32328	4.14853E-07	9.29656E-06
4036	*LRP2*	-216413.1787	-216400.3761	0.24341	0.27293	4.18917E-07	9.29656E-06
7060	*THBS4*	-34428.8437	-34417.05884	0.07299	0.10075	1.20469E-06	2.36667E-05
7049	*TGFBR3*	-34458.47132	-34448.1189	0.17758	0.23034	5.35803E-06	7.92833E-05
30812	*SOX8*	-14568.28338	-14557.97499	0.04064	0.06628	5.61019E-06	0.000079662
55717	*WDR11*	-41057.77432	-41047.90562	0.06026	0.08025	8.88452E-06	0.000108703
139285	*AMER1*	-46708.2831	-46698.95823	0.33785	0.41649	1.57069E-05	0.00017979
5925	*RB1*	-24000.09847	-23991.00391	0.11006	0.14979	2.00019E-05	0.000221875
1848	*DUSP6*	-9329.832217	-9321.045935	0.0205	0.03838	2.7655E-05	0.000289071
6535	*SLC6A8*	-14870.95167	-14863.0564	0.02864	0.04581	7.07558E-05	0.000644462
58	*ACTA1*	-8343.146936	-8336.512655	0.00248	0.00688	0.000269894	0.002039201
51715	*RAB23*	-6153.357213	-6146.733378	0.05758	0.10496	0.000272919	0.002039201
654231	*OCM*	-3029.218817	-3022.604562	0.04575	0.10814	0.000275723	0.002039201
6206	*RPS12*	-2803.98049	-2797.607403	0.0018	0.0192	0.000356738	0.00253284

5168	*ENPP2*	-28451.65225	-28445.35128	0.0714	0.09266	0.000385349	0.002636517
64895	*PAPOLG*	-19332.71545	-19326.41653	0.0692	0.09407	0.000386194	0.002636517
10371	*SEMA3A*	-22535.43741	-22529.24057	0.05339	0.07236	0.000430792	0.002832059
9743	*ARHGAP32*	-85413.13016	-85407.09885	0.18369	0.20904	0.000514432	0.003261131
123722	*FSD2*	-33814.55283	-33808.60741	0.1786	0.22125	0.000564108	0.00345273
367	*AR*	-28556.41935	-28550.49738	0.1256	0.15887	0.00057849	0.003480745
3488	*IGFBP5*	-7328.641895	-7323.192662	0.03843	0.06174	0.000962439	0.005338529
3973	*LHCGR*	-23868.73685	-23863.37486	0.15242	0.19155	0.001057558	0.00568838
1793	*DOCK1*	-62877.83514	-62872.59063	0.03039	0.0374	0.001200866	0.006362797
5894	*RAF1*	-17163.96241	-17158.76799	0.02469	0.03675	0.001267798	0.006618651
412	*STS*	-21264.5389	-21259.47067	0.13669	0.17594	0.001453644	0.007372052
114569	*MAL2*	-5834.008203	-5829.149875	0.14317	0.22734	0.001826055	0.008991537
3236	*HOXD10*	-4267.384622	-4262.53775	0.0667	0.12882	0.001848964	0.008991537

Table S4. Rapidly evolving genes in a 62-species mammalian data set.

Gene	lnL (one ratio)	lnL (two ratio)	ω in CDT species	ω in (IDT+UDT) species	p value
RAB2B	-7138.539649	-7106.814199	0.10229	0.28707	1.67E-15
RXFP1	-34104.06537	-34082.05865	0.21661	0.32197	3.26E-11
WDR11	-45060.18142	-45038.55305	0.05436	0.08245	4.80E-11
CHD7	-95002.07181	-94984.4701	0.06972	0.09033	2.97E-09
SNRPN	-2824.643225	-2807.714075	0.01402	0.1135	5.93E-09
PTCH1	-47730.7798	-47715.03224	0.04315	0.06272	2.00E-08
FGD1	-26477.64314	-26462.11534	0.06493	0.10289	2.51E-08
RXFP2	-30575.59361	-30560.48522	0.22723	0.32544	3.86E-08
RAB3GAP2	-61502.5088	-61488.47334	0.113	0.14484	1.17E-07
LRP2	-248208.502	-248196.2839	0.24637	0.27432	7.68E-07
RB1	-26864.63077	-26852.89255	0.10615	0.14924	1.26E-06
THBS4	-37634.66801	-37623.6388	0.07098	0.09672	2.64E-06
DUSP6	-10479.71461	-10469.44653	0.01951	0.03758	5.85E-06
PAPOLG	-21720.61858	-21710.91475	0.06761	0.09733	1.06E-05
ARHGAP32	-96744.82089	-96735.2526	0.17887	0.20921	1.22E-05
IGFBP5	-8418.074797	-8408.941054	0.03793	0.06757	1.92E-05
TGFBR3	-40037.56496	-40028.98982	0.18616	0.23339	3.45E-05
ADAMTS20	-87238.48954	-87230.8115	0.19161	0.22087	8.90E-05
AMER1	-50837.87619	-50830.70224	0.34016	0.40614	0.000151949
STS	-50837.87619	-50830.70224	0.34016	0.40614	0.000151949
SEMA3A	-26019.37648	-26013.05149	0.05331	0.07166	0.00037557
FSD2	-35909.75911	-35904.1219	0.17322	0.21457	0.000785821
ENPP2	-32552.60986	-32547.19505	0.07272	0.09174	0.000998895
SOX8	-11989.95885	-11984.92132	0.04329	0.064	0.001502896
RAB23	-7119.775777	-7114.842339	0.06327	0.10368	0.00168281
DOCK1	-69921.01352	-69916.52194	0.02987	0.03603	0.002724799
SLC6A8	-16434.39816	-16429.93412	0.03061	0.04283	0.002808188
OCM	-3303.107196	-3299.003443	0.04802	0.09095	0.004171745
LHCGR	-25829.76244	-25825.684	0.1527	0.18609	0.004289839

HOXD10	-4708.342021	-4704.363166	0.07202	0.12697	0.004788296
ACTA1	-9200.109361	-9196.136488	0.00236	0.00538	0.004820054
GLI2	-63175.83986	-63172.06272	0.11011	0.12443	0.005986731
AR	-31930.4169	-31926.88759	0.12749	0.15193	0.007888536
RAF1	-19516.81014	-19515.07232	0.02874	0.03564	0.062278551
MAL2	-5586.44656	-5584.953648	0.16133	0.21141	0.083996525
RPS12	-3281.663943	-3281.507895	0.01291	0.01886	0.576396835

Table S5. KEGG enrichment of rapidly evolving genes in ascrotal IDT and UDT mammals.

ID	Description	GeneRatio	BgRatio	pvalue	p.adjust	qvalue	geneID	Count
hsa04340	Hedgehog signaling pathway	3/19	47/7847	0.00018247	0.01788208	0.01478969	2736/5727/4036	3

Table S6. GO enrichment of rapidly evolving genes in ascrotal IDT and UDT mammals.

ONTOLOGY	ID	Description	GeneRatio	BgRatio	pvalue	p.adjust	qvalue	geneID	Count
BP	GO:0048608	reproductive structure development	9/33	429/17381	7.13E-08	4.77E-05	3.02E-05	2736/5727/55636/122042/4036/30812/10371/367/3973	9
BP	GO:0061458	reproductive system development	9/33	432/17381	7.57E-08	4.77E-05	3.02E-05	2736/5727/55636/122042/4036/30812/10371/367/3973	9
BP	GO:0008584	male gonad development	6/33	139/17381	2.18E-07	7.15E-05	4.54E-05	122042/4036/30812/10371/367/3973	6
BP	GO:0046546	development of primary male sexual characteristics	6/33	140/17381	2.27E-07	7.15E-05	4.54E-05	122042/4036/30812/10371/367/3973	6
BP	GO:0001708	cell fate specification	5/33	83/17381	4.70E-07	9.67E-05	6.13E-05	2736/5727/1848/367/3236	5
BP	GO:0060537	muscle tissue development	8/33	387/17381	4.79E-07	9.67E-05	6.13E-05	55636/4036/7049/30812/5925/58/3488/3236	8
BP	GO:0046661	male sex differentiation	6/33	162/17381	5.37E-07	9.67E-05	6.13E-05	122042/4036/30812/10371/367/3973	6
BP	GO:0007548	sex differentiation	7/33	271/17381	6.26E-07	9.86E-05	6.25E-05	55636/122042/4036/30812/10371/367/3973	7
BP	GO:0060443	mammary gland morphogenesis	4/33	45/17381	1.52E-06	0.00021258	0.00013479	2736/5727/367/3488	4
BP	GO:0048806	genitalia development	4/33	47/17381	1.81E-06	0.00022842	0.00014484	55636/4036/367/3973	4
BP	GO:0003002	regionalization	7/33	332/17381	2.43E-06	0.00027803	0.0001763	2736/5727/4036/1848/10371/367/3236	7

BP	GO:0022612	gland morphogenesis	5/33	119/17381	2.81E-06	0.00029399	0.00018641	2736/5727/10371/367/3488	5
BP	GO:0008406	gonad development	6/33	218/17381	3.03E-06	0.00029399	0.00018641	122042/4036/30812/10371/367/3973	6
BP	GO:0045137	development of primary sexual characteristics	6/33	224/17381	3.55E-06	0.00031931	0.00020247	122042/4036/30812/10371/367/3973	6
BP	GO:0014706	striated muscle tissue development	7/33	369/17381	4.87E-06	0.00040939	0.00025959	55636/4036/7049/30812/5925/58/3236	7
BP	GO:0007517	muscle organ development	7/33	389/17381	6.89E-06	0.00052923	0.00033558	55636/4036/7049/30812/5925/58/3236	7
BP	GO:0045165	cell fate commitment	6/33	253/17381	7.14E-06	0.00052923	0.00033558	2736/5727/30812/1848/367/3236	6
BP	GO:0016331	morphogenesis of embryonic epithelium	5/33	148/17381	8.19E-06	0.00057344	0.00036361	2736/5727/4036/30812/367	5
BP	GO:0007389	pattern specification process	7/33	422/17381	1.17E-05	0.00073763	0.00046772	2736/5727/4036/1848/10371/367/3236	7
BP	GO:0048732	gland development	7/33	422/17381	1.17E-05	0.00073763	0.00046772	2736/5727/7049/10371/367/3488/5894	7
BP	GO:0061138	morphogenesis of a branching epithelium	5/33	180/17381	2.11E-05	0.00126826	0.00080418	2736/5727/30812/10371/367	5
BP	GO:0048638	regulation of developmental growth	6/33	310/17381	2.26E-05	0.00129637	0.00082201	5727/55636/7049/1848/10371/367	6
BP	GO:0060603	mammary gland duct morphogenesis	3/33	31/17381	2.70E-05	0.00148082	0.00093897	2736/5727/367	3
BP	GO:0001763	morphogenesis of a branching structure	5/33	193/17381	2.95E-05	0.00150135	0.00095198	2736/5727/30812/10371/367	5

BP	GO:0021522	spinal cord motor neuron differentiation	3/33	32/17381	2.98E-05	0.00150135	0.00095198	2736/5727/3236	3
BP	GO:0035239	tube morphogenesis	6/33	347/17381	4.26E-05	0.0020639	0.00130869	2736/5727/55636/4036/30812/367	6
BP	GO:0035137	hindlimb morphogenesis	3/33	37/17381	4.64E-05	0.00216365	0.00137194	5727/55636/3236	3
BP	GO:0050673	epithelial cell proliferation	6/33	361/17381	5.31E-05	0.0023886	0.00151458	5727/7060/7049/5925/367/3488	6
BP	GO:0021517	ventral spinal cord development	3/33	44/17381	7.83E-05	0.00340265	0.00215757	2736/5727/3236	3
BP	GO:0060485	mesenchyme development	5/33	246/17381	9.36E-05	0.0036043	0.00228543	7049/30812/139285/58/10371	5
BP	GO:0030326	embryonic limb morphogenesis	4/33	127/17381	9.44E-05	0.0036043	0.00228543	2736/5727/55636/3236	4
BP	GO:0035113	embryonic appendage morphogenesis	4/33	127/17381	9.44E-05	0.0036043	0.00228543	2736/5727/55636/3236	4
BP	GO:0050680	negative regulation of epithelial cell proliferation	4/33	127/17381	9.44E-05	0.0036043	0.00228543	5727/7049/5925/367	4
BP	GO:0030850	prostate gland development	3/33	48/17381	0.00010175	0.00366292	0.0023226	2736/5727/367	3
BP	GO:0055010	ventricular cardiac muscle tissue morphogenesis	3/33	48/17381	0.00010175	0.00366292	0.0023226	55636/4036/7049	3
BP	GO:0030879	mammary gland development	4/33	131/17381	0.00010645	0.00372583	0.00236249	2736/5727/367/3488	4
BP	GO:0021515	cell differentiation in spinal cord	3/33	52/17381	0.00012934	0.00440447	0.00279281	2736/5727/3236	3

BP	GO:0003229	ventricular cardiac muscle tissue development	3/33	53/17381	0.00013692	0.00454001	0.00287875	55636/4036/7049	3
BP	GO:0060513	prostatic bud formation	2/33	10/17381	0.00015582	0.00503414	0.00319207	2736/367	2
BP	GO:0035107	appendage morphogenesis	4/33	147/17381	0.00016606	0.00510336	0.00323597	2736/5727/55636/3236	4
BP	GO:0035108	limb morphogenesis	4/33	147/17381	0.00016606	0.00510336	0.00323597	2736/5727/55636/3236	4
BP	GO:0048754	branching morphogenesis of an epithelial tube	4/33	148/17381	0.00017045	0.00511341	0.00324234	2736/5727/30812/367	4
BP	GO:0048645	animal organ formation	3/33	60/17381	0.00019821	0.00580796	0.00368274	2736/4036/367	3
BP	GO:0007519	skeletal muscle tissue development	4/33	158/17381	0.00021903	0.0062723	0.00397717	30812/5925/58/3236	4
BP	GO:0055008	cardiac muscle tissue morphogenesis	3/33	64/17381	0.00024008	0.00636011	0.00403285	55636/4036/7049	3
BP	GO:0061180	mammary gland epithelium development	3/33	66/17381	0.000263	0.00636011	0.00403285	2736/5727/367	3
BP	GO:0050678	regulation of epithelial cell proliferation	5/33	307/17381	0.00026316	0.00636011	0.00403285	5727/7060/7049/5925/367	5
BP	GO:0060538	skeletal muscle organ development	4/33	166/17381	0.00026454	0.00636011	0.00403285	30812/5925/58/3236	4
BP	GO:0021520	spinal cord motor neuron cell fate specification	2/33	13/17381	0.00026912	0.00636011	0.00403285	2736/3236	2
BP	GO:0042659	regulation of cell fate specification	2/33	13/17381	0.00026912	0.00636011	0.00403285	1848/367	2

BP	GO:0043568	positive regulation of insulin-like growth factor receptor signaling pathway	2/33	13/17381	0.00026912	0.00636011	0.00403285	367/3488	2
BP	GO:0060601	lateral sprouting from an epithelium	2/33	13/17381	0.00026912	0.00636011	0.00403285	2736/367	2
BP	GO:0060411	cardiac septum morphogenesis	3/33	67/17381	0.00027498	0.00636011	0.00403285	55636/4036/7049	3
BP	GO:0060562	epithelial tube morphogenesis	5/33	310/17381	0.00027525	0.00636011	0.00403285	2736/5727/4036/30812/367	5
BP	GO:0021953	central nervous system neuron differentiation	4/33	171/17381	0.00029621	0.00636011	0.00403285	2736/5727/10371/3236	4
BP	GO:0001701	in utero embryonic development	5/33	315/17381	0.00029633	0.00636011	0.00403285	2736/5727/55636/30812/367	5
BP	GO:0008589	regulation of smoothened signaling pathway	3/33	69/17381	0.00029997	0.00636011	0.00403285	2736/5727/5925	3
BP	GO:0001655	urogenital system development	5/33	316/17381	0.00030069	0.00636011	0.00403285	2736/5727/30812/139285/367	5
BP	GO:0048736	appendage development	4/33	172/17381	0.00030286	0.00636011	0.00403285	2736/5727/55636/3236	4
BP	GO:0060173	limb development	4/33	172/17381	0.00030286	0.00636011	0.00403285	2736/5727/55636/3236	4
BP	GO:0060831	smoothened signaling pathway involved in dorsal/ventral neural tube patterning	2/33	14/17381	0.00031361	0.00647775	0.00410744	2736/5727	2
BP	GO:0003208	cardiac ventricle morphogenesis	3/33	72/17381	0.00034013	0.00691232	0.004383	55636/4036/7049	3
BP	GO:0060415	muscle tissue morphogenesis	3/33	77/17381	0.00041449	0.00828985	0.00525647	55636/4036/7049	3

BP	GO:0048762	mesenchymal cell differentiation	4/33	190/17381	0.00044175	0.00869692	0.00551459	7049/30812/139285/10371	4
BP	GO:0060572	morphogenesis of an epithelial bud	2/33	17/17381	0.00046702	0.00891578	0.00565336	2736/367	2
BP	GO:0060977	coronary vasculature morphogenesis	2/33	17/17381	0.00046702	0.00891578	0.00565336	4036/7049	2
BP	GO:0030819	positive regulation of cAMP biosynthetic process	3/33	83/17381	0.00051661	0.00944587	0.00598948	122042/3973/5894	3
BP	GO:0048644	muscle organ morphogenesis	3/33	83/17381	0.00051661	0.00944587	0.00598948	55636/4036/7049	3
BP	GO:0030540	female genitalia development	2/33	18/17381	0.00052477	0.00944587	0.00598948	55636/4036	2
BP	GO:0072337	modified amino acid transport	2/33	18/17381	0.00052477	0.00944587	0.00598948	4036/6535	2
BP	GO:0001649	osteoblast differentiation	4/33	207/17381	0.00061001	0.01082555	0.00686432	2736/5727/30812/3488	4
BP	GO:0009612	response to mechanical stimulus	4/33	210/17381	0.00064384	0.01126721	0.00714437	2736/5727/58/5894	4
BP	GO:0030816	positive regulation of cAMP metabolic process	3/33	92/17381	0.00069793	0.01204645	0.00763848	122042/3973/5894	3
BP	GO:0009953	dorsal/ventral pattern formation	3/33	95/17381	0.00076629	0.01304762	0.00827331	2736/5727/1848	3
BP	GO:0003279	cardiac septum development	3/33	98/17381	0.00083878	0.01390602	0.0088176	55636/4036/7049	3
BP	GO:0021510	spinal cord development	3/33	98/17381	0.00083878	0.01390602	0.0088176	2736/5727/3236	3

BP	GO:0030539	male genitalia development	2/33	23/17381	0.00086262	0.0141156	0.00895049	367/3973	2
BP	GO:0030804	positive regulation of cyclic nucleotide biosynthetic process	3/33	101/17381	0.00091549	0.01478864	0.00937726	122042/3973/5894	3
BP	GO:0021904	dorsal/ventral neural tube patterning	2/33	24/17381	0.00093992	0.01499118	0.00950569	2736/5727	2
BP	GO:0003007	heart morphogenesis	4/33	235/17381	0.00097957	0.0154282	0.0097828	5727/55636/4036/7049	4
BP	GO:0043501	skeletal muscle adaptation	2/33	25/17381	0.00102044	0.01567999	0.00994245	58/3488	2
BP	GO:0060384	innervation	2/33	25/17381	0.00102044	0.01567999	0.00994245	55636/10371	2
BP	GO:0043567	regulation of insulin-like growth factor receptor signaling pathway	2/33	26/17381	0.00110417	0.01636769	0.01037851	367/3488	2
BP	GO:0060571	morphogenesis of an epithelial fold	2/33	26/17381	0.00110417	0.01636769	0.01037851	2736/367	2
BP	GO:0060740	prostate gland epithelium morphogenesis	2/33	26/17381	0.00110417	0.01636769	0.01037851	2736/367	2
BP	GO:0030810	positive regulation of nucleotide biosynthetic process	3/33	109/17381	0.00114143	0.016531	0.01048206	122042/3973/5894	3
BP	GO:1900373	positive regulation of purine nucleotide biosynthetic process	3/33	109/17381	0.00114143	0.016531	0.01048206	122042/3973/5894	3
BP	GO:0045879	negative regulation of smoothened signaling pathway	2/33	27/17381	0.00119109	0.01705422	0.01081383	5727/5925	2
BP	GO:0030801	positive regulation of cyclic nucleotide metabolic process	3/33	112/17381	0.00123443	0.01747623	0.01108142	122042/3973/5894	3

BP	GO:0060512	prostate gland morphogenesis	2/33	28/17381	0.00128119	0.01793665	0.01137336	2736/367	2
BP	GO:0034698	response to gonadotropin	2/33	29/17381	0.00137446	0.01903099	0.01206727	7049/3973	2
BP	GO:0046660	female sex differentiation	3/33	117/17381	0.00139983	0.01917158	0.01215642	55636/4036/3973	3
BP	GO:0003231	cardiac ventricle development	3/33	119/17381	0.0014697	0.01930543	0.01224129	55636/4036/7049	3
BP	GO:0010092	specification of animal organ identity	2/33	30/17381	0.00147089	0.01930543	0.01224129	4036/367	2
BP	GO:0021772	olfactory bulb development	2/33	30/17381	0.00147089	0.01930543	0.01224129	55636/10371	2
BP	GO:0031128	developmental induction	2/33	30/17381	0.00147089	0.01930543	0.01224129	30812/367	2
BP	GO:0001822	kidney development	4/33	264/17381	0.00150532	0.01955359	0.01239864	2736/5727/30812/139285	4
BP	GO:0009954	proximal/distal pattern formation	2/33	31/17381	0.00157047	0.01969291	0.01248698	2736/3236	2
BP	GO:0021988	olfactory lobe development	2/33	31/17381	0.00157047	0.01969291	0.01248698	55636/10371	2
BP	GO:0001838	embryonic epithelial tube formation	3/33	122/17381	0.00157856	0.01969291	0.01248698	5727/4036/30812	3
BP	GO:0003206	cardiac chamber morphogenesis	3/33	122/17381	0.00157856	0.01969291	0.01248698	55636/4036/7049	3
BP	GO:0090130	tissue migration	4/33	269/17381	0.00161255	0.01976779	0.01253446	58/5168/10371/1793	4

BP	GO:0007224	smoothened signaling pathway	3/33	123/17381	0.00161594	0.01976779	0.01253446	2736/5727/5925	3
BP	GO:0048665	neuron fate specification	2/33	33/17381	0.00177901	0.02155344	0.01366672	2736/3236	2
BP	GO:0030817	regulation of cAMP biosynthetic process	3/33	129/17381	0.00185191	0.02188485	0.01387686	122042/3973/5894	3
BP	GO:0072001	renal system development	4/33	280/17381	0.00186706	0.02188485	0.01387686	2736/5727/30812/139285	4
BP	GO:0061384	heart trabecula morphogenesis	2/33	34/17381	0.00188796	0.02188485	0.01387686	55636/7049	2
BP	GO:1905332	positive regulation of morphogenesis of an epithelium	2/33	34/17381	0.00188796	0.02188485	0.01387686	30812/367	2
BP	GO:0072175	epithelial tube formation	3/33	130/17381	0.00189321	0.02188485	0.01387686	5727/4036/30812	3
BP	GO:0006171	cAMP biosynthetic process	3/33	132/17381	0.00197754	0.02265186	0.01436321	122042/3973/5894	3
BP	GO:0007190	activation of adenylate cyclase activity	2/33	36/17381	0.00211515	0.02361952	0.01497679	3973/5894	2
BP	GO:0060612	adipose tissue development	2/33	36/17381	0.00211515	0.02361952	0.01497679	30812/139285	2
BP	GO:0010720	positive regulation of cell development	5/33	488/17381	0.00211826	0.02361952	0.01497679	2736/4036/30812/10371/1793	5
BP	GO:0001501	skeletal system development	5/33	491/17381	0.00217563	0.02404647	0.01524751	2736/55636/139285/51715/3236	5
BP	GO:0010453	regulation of cell fate commitment	2/33	37/17381	0.00223337	0.02446992	0.01551601	1848/367	2

BP	GO:0001755	neural crest cell migration	2/33	38/17381	0.00235465	0.02557635	0.01621759	30812/10371		2
BP	GO:0048009	insulin-like growth factor receptor signaling pathway	2/33	39/17381	0.00247899	0.02669678	0.01692803	367/3488		2
BP	GO:0035148	tube formation	3/33	144/17381	0.00253286	0.02698081	0.01710813	5727/4036/30812		3
BP	GO:0045981	positive regulation of nucleotide metabolic process	3/33	145/17381	0.00258305	0.02698081	0.01710813	122042/3973/5894		3
BP	GO:1900544	positive regulation of purine nucleotide metabolic process	3/33	145/17381	0.00258305	0.02698081	0.01710813	122042/3973/5894		3
BP	GO:0021532	neural tube patterning	2/33	40/17381	0.00260637	0.02698081	0.01710813	2736/5727		2
BP	GO:0030814	regulation of cAMP metabolic process	3/33	146/17381	0.00263384	0.02698081	0.01710813	122042/3973/5894		3
BP	GO:0035264	multicellular organism growth	3/33	146/17381	0.00263384	0.02698081	0.01710813	5727/55636/367		3
BP	GO:0030802	regulation of cyclic nucleotide biosynthetic process	3/33	149/17381	0.00278994	0.02834934	0.0179759	122042/3973/5894		3
BP	GO:0045668	negative regulation of osteoblast differentiation	2/33	42/17381	0.00287022	0.02893181	0.01834523	5727/3488		2
BP	GO:0048483	autonomic nervous system development	2/33	43/17381	0.00300667	0.03006666	0.01906482	30812/10371		2
BP	GO:0007188	adenylate cyclase-modulating G-protein coupled receptor signaling pathway	3/33	155/17381	0.00311902	0.03094463	0.01962153	59350/122042/3973		3
BP	GO:0003205	cardiac chamber development	3/33	156/17381	0.00317609	0.03126463	0.01982444	55636/4036/7049		3

BP	GO:0060976	coronary vasculature development	2/33	45/17381	0.00328854	0.0319085	0.02023271	4036/7049	2
BP	GO:0021915	neural tube development	3/33	158/17381	0.00329215	0.0319085	0.02023271	2736/5727/4036	3
BP	GO:1900371	regulation of purine nucleotide biosynthetic process	3/33	159/17381	0.00335114	0.03223236	0.02043806	122042/3973/5894	3
BP	GO:0030808	regulation of nucleotide biosynthetic process	3/33	160/17381	0.00341079	0.03255749	0.02064423	122042/3973/5894	3
BP	GO:0046058	cAMP metabolic process	3/33	162/17381	0.00353202	0.03346126	0.02121729	122042/3973/5894	3
BP	GO:0045762	positive regulation of adenylate cyclase activity	2/33	47/17381	0.00358233	0.03354047	0.02126751	3973/5894	2
BP	GO:0052652	cyclic purine nucleotide metabolic process	3/33	163/17381	0.00359362	0.03354047	0.02126751	122042/3973/5894	3
BP	GO:0009190	cyclic nucleotide biosynthetic process	3/33	164/17381	0.00365588	0.03387062	0.02147686	122042/3973/5894	3
BP	GO:2000179	positive regulation of neural precursor cell proliferation	2/33	48/17381	0.00373366	0.0342862	0.02174037	2736/4036	2
BP	GO:0048469	cell maturation	3/33	166/17381	0.00378237	0.0342862	0.02174037	122042/30812/5925	3
BP	GO:0048771	tissue remodeling	3/33	166/17381	0.00378237	0.0342862	0.02174037	55636/7060/3488	3
BP	GO:0021545	cranial nerve development	2/33	49/17381	0.00388793	0.03470775	0.02200767	55636/10371	2
BP	GO:0061383	trabecula morphogenesis	2/33	49/17381	0.00388793	0.03470775	0.02200767	55636/7049	2

BP	GO:0035265	organ growth	3/33	168/17381	0.00391151	0.03470775	0.02200767	7049/1848/367	3
BP	GO:0060324	face development	2/33	50/17381	0.00404514	0.03564247	0.02260036	55636/5894	2
BP	GO:0030799	regulation of cyclic nucleotide metabolic process	3/33	171/17381	0.00411023	0.03596454	0.02280458	122042/3973/5894	3
BP	GO:0001667	ameboidal-type cell migration	4/33	353/17381	0.00429897	0.03735657	0.02368725	30812/5168/10371/1793	4
BP	GO:0035904	aorta development	2/33	52/17381	0.0043683	0.03769903	0.0239044	55636/4036	2
BP	GO:0060688	regulation of morphogenesis of a branching structure	2/33	53/17381	0.00453424	0.03886489	0.02464365	30812/367	2
BP	GO:0014015	positive regulation of gliogenesis	2/33	54/17381	0.00470306	0.03977086	0.02521812	4036/30812	2
BP	GO:0061005	cell differentiation involved in kidney development	2/33	54/17381	0.00470306	0.03977086	0.02521812	5727/139285	2
BP	GO:0014888	striated muscle adaptation	2/33	55/17381	0.00487477	0.04028043	0.02554123	58/3488	2
BP	GO:2000243	positive regulation of reproductive process	2/33	55/17381	0.00487477	0.04028043	0.02554123	10371/367	2
BP	GO:0030900	forebrain development	4/33	366/17381	0.0048859	0.04028043	0.02554123	2736/55636/4036/10371	4
BP	GO:0007187	G-protein coupled receptor signaling pathway, coupled to cyclic nucleotide second messenger	3/33	182/17381	0.0048912	0.04028043	0.02554123	59350/122042/3973	3
BP	GO:0001503	ossification	4/33	373/17381	0.00522333	0.0427363	0.02709845	2736/5727/30812/3488	4

BP	GO:0007565	female pregnancy	3/33	190/17381	0.00551189	0.04480635	0.02841105	367/3488/412	3
BP	GO:0001658	branching involved in ureteric bud morphogenesis	2/33	59/17381	0.00559014	0.04515109	0.02862964	5727/30812	2
BP	GO:0031281	positive regulation of cyclase activity	2/33	60/17381	0.00577606	0.04606228	0.02920741	3973/5894	2
BP	GO:0051349	positive regulation of lyase activity	2/33	60/17381	0.00577606	0.04606228	0.02920741	3973/5894	2
BP	GO:0014032	neural crest cell development	2/33	61/17381	0.00596481	0.04726826	0.02997211	30812/10371	2
BP	GO:0060541	respiratory system development	3/33	197/17381	0.00609227	0.04797661	0.03042126	2736/55636/3488	3
MF	GO:0016500	protein-hormone receptor activity	2/36	12/17354	0.00027256	0.02491408	0.01794366	122042/3973	2
MF	GO:0042562	hormone binding	3/36	65/17354	0.00032782	0.02491408	0.01794366	122042/367/3973	3

Table S7. Rapidly evolving genes in UDT mammals.

EntrezID	Gene	lnL (one ratio)	lnL (two ratio)	*ω* in IDT mammals	*ω* in UDT mammals	p value	p adjust (<0.01)
84932	*RAB2B*	-4221.354703	-4173.474307	0.1008	0.65038	0	0.00E+00
6638	*SNRPN*	-2918.539197	-2880.061522	0.01179	0.29374	0	0.00E+00
114788	*CSMD3*	-55501.95691	-55469.1497	0.03008	0.05122	5.55E-16	3.94E-14
10371	*SEMA3A*	-12579.51457	-12555.3777	0.04336	0.10183	3.71E-12	1.88E-10
80070	*ADAMTS20*	-41397.54195	-41373.61586	0.18603	0.27345	4.60E-12	2.04E-10
5925	*RB1*	-14047.49524	-14024.35823	0.10615	0.20681	1.03E-11	4.06E-10
83860	*TAF3*	-19567.40854	-19546.66684	0.08858	0.16625	1.19E-10	3.84E-09
55636	*CHD7*	-48305.36425	-48285.79098	0.07345	0.11136	3.93E-10	1.16E-08
2736	*GLI2*	-35966.63147	-35947.45974	0.10782	0.15715	5.93E-10	1.50E-08
59350	*RXFP1*	-16720.3224	-16702.90797	0.2351	0.40194	3.60E-09	8.52E-08
122042	*RXFP2*	-15156.78141	-15140.49806	0.23559	0.41406	1.15E-08	2.55E-07
10659	*CELF2*	-5410.680587	-5394.798329	0.00758	0.04529	1.74E-08	3.63E-07
7879	*RAB7A*	-2683.671541	-2668.648228	0.00855	0.06095	4.22E-08	7.88E-07
6873	*TAF2*	-19517.78661	-19506.71719	0.03105	0.05131	2.54E-06	4.29E-05
2625	*GATA3*	-6706.262785	-6696.326791	0.01768	0.04104	8.28E-06	1.34E-04
654231	*OCM*	-1697.71463	-1687.847055	0.04018	0.19238	8.90E-06	1.37E-04
2195	*FAT1*	-104124.2598	-104114.8529	0.08878	0.10648	1.44E-05	2.04E-04
9743	*ARHGAP32*	-46456.58304	-46447.3288	0.19328	0.24368	1.69E-05	2.31E-04
7514	*XPO1*	-11705.2537	-11696.07708	0.00287	0.01222	1.84E-05	2.42E-04
546	*ATRX*	-40131.8264	-40123.006	0.12339	0.15963	2.67E-05	3.39E-04
54780	*NSMCE4A*	-7117.481841	-7109.254512	0.1399	0.23388	4.98E-05	5.70E-04
3623	*INHA*	-9694.129071	-9686.218964	0.19942	0.30977	6.97E-05	7.73E-04
5727	*PTCH1*	-23618.77152	-23610.90046	0.05259	0.07746	7.26E-05	7.81E-04

3688	*ITGB1*	-13369.60215	-13361.79851	0.03271	0.05493	7.80E-05	8.14E-04
6657	*SOX2*	-2941.685215	-2934.11051	0.0074	0.02623	9.93E-05	1.01E-03
2245	*FGD1*	-15671.57945	-15664.12792	0.09735	0.14746	0.000113179	1.09E-03
3235	*HOXD9*	-4169.941113	-4162.719495	0.07784	0.15864	0.000144448	1.31E-03
53335	*BCL11A*	-6671.393023	-6664.218968	0.01685	0.04837	0.000151932	1.35E-03
7549	*ZNF2*	-11213.2664	-11206.17843	0.1879	0.27829	0.000166485	1.44E-03
9968	*MED12*	-32013.5064	-32007.22478	0.03483	0.05151	0.000393408	3.04E-03
3207	*HOXA11*	-3048.398599	-3042.545744	0.0272	0.07104	0.000623086	4.42E-03
990	*CDC6*	-12093.8966	-12088.2624	0.22923	0.32933	0.000788379	5.28E-03
9712	*USP6NL*	-18755.18317	-18749.7515	0.17686	0.24136	0.000980867	6.16E-03
5894	*RAF1*	-9795.256921	-9789.921442	0.02467	0.04186	0.001088307	6.51E-03
825	*CAPN3*	-14641.14572	-14635.85695	0.0726	0.1051	0.001144699	6.66E-03
55717	*WDR11*	-23303.61276	-23298.35408	0.06368	0.08481	0.001182592	6.77E-03
4692	*NDN*	-6107.253238	-6102.049926	0.09536	0.15162	0.001255641	7.08E-03
79892	*MCMBP*	-12040.90329	-12035.75078	0.05217	0.07564	0.001326691	7.36E-03
3204	*HOXA7*	-3630.349491	-3625.229677	0.08172	0.15763	0.001374553	7.51E-03
5216	*PFN1*	-1952.873625	-1947.811321	0.11303	0.3557	0.001463028	7.87E-03
1875	*E2F5*	-4696.672731	-4691.682834	0.08161	0.15038	0.001582674	8.05E-03
50810	*HDGFL3*	-2205.113221	-2200.178927	0.01454	0.04668	0.001681245	8.29E-03
53346	*TM6SF1*	-6781.829155	-6776.97764	0.09651	0.16036	0.001839644	8.83E-03
3236	*HOXD10*	-3187.904488	-3183.103776	0.05684	0.12812	0.001944265	9.08E-03
10114	*HIPK3*	-18413.30248	-18408.54041	0.11785	0.15992	0.002027879	9.35E-03
4853	*NOTCH2*	-45155.40415	-45150.67454	0.06893	0.08522	0.002100915	9.56E-03

Table S8. GO enrichment of genes evolved with significant regression with testicular descent.

ONTOLOGY	ID	Description	GeneRatio	BgRatio	pvalue	p.adjust	qvalue	geneID	Count
BP	GO:0034086	maintenance of sister chromatid cohesion	2/12	12/17381	2.87E-05	0.00359118	0.00235884	546/79075	2
BP	GO:0034088	maintenance of mitotic sister chromatid cohesion	2/12	12/17381	2.87E-05	0.00359118	0.00235884	546/79075	2
BP	GO:0007064	mitotic sister chromatid cohesion	2/12	23/17381	0.00010967	0.00913881	0.00600276	546/79075	2
BP	GO:0090329	regulation of DNA-dependent DNA replication	2/12	46/17381	0.00044469	0.02779322	0.01825576	546/79075	2

Table S9. GO enrichment of positively selected genes in ascrotal IDT and UDT mammals.

ONTOLOGY	ID	Description	GeneRatio	BgRatio	pvalue	p.adjust	qvalue	geneID	Count
CC	GO:0005884	actin filament	2/12	88/18511	0.00142974	0.04429945	0.02569463	2316/58	2
CC	GO:0030017	sarcomere	2/12	192/18511	0.00659675	0.04429945	0.02569463	2316/58	2
CC	GO:0042555	MCM complex	1/12	11/18511	0.00710974	0.04429945	0.02569463	79892	1
CC	GO:0017101	aminoacyl-tRNA synthetase multienzyme complex	1/12	12/18511	0.00775378	0.04429945	0.02569463	7965	1
CC	GO:0044449	contractile fiber part	2/12	209/18511	0.00777228	0.04429945	0.02569463	2316/58	2
CC	GO:0030016	myofibril	2/12	213/18511	0.00806174	0.04429945	0.02569463	2316/58	2
CC	GO:0032045	guanyl-nucleotide exchange factor complex	1/12	13/18511	0.00839743	0.04429945	0.02569463	1793	1
CC	GO:0043240	Fanconi anaemia nuclear complex	1/12	13/18511	0.00839743	0.04429945	0.02569463	2178	1
CC	GO:0043292	contractile fiber	2/12	224/18511	0.00888275	0.04429945	0.02569463	2316/58	2
CC	GO:0097440	apical dendrite	1/12	14/18511	0.0090407	0.04429945	0.02569463	2316	1
CC	GO:0031143	pseudopodium	1/12	17/18511	0.01096822	0.04885844	0.02833895	5894	1

Table S10. The observed convergent/parallel amino acid substitutions in the ascrotal IDT and UDT branches.

Gene	EntrezID	Ascrotal_branch_pair	Number_of observed substitution	Number_of expected substitution	P_value (Poisson_test)
ADAMTS20	80070	66-pteVam1	1	0.2586	0.22786819
ADAMTS20	80070	96-94	1	0.3097	0.26633298
ADAMTS20	80070	97-loxAfr3	4	3.8912	0.79947306
ADAMTS20	80070	odoRosDiv1-turTru2	1	0.1567	0.14503949
ADAMTS20	80070	pteAle1-58	1	0.5195	0.40518212
ADAMTS20	80070	pteVam1-57	1	0.3637	0.30490031
ADAMTS20	80070	pteVam1-58	1	0.5265	0.4093313
ADAMTS20	80070	turTru2-hetGla2	1	0.5633	0.43067282
AFTPH	54812	66-58	2	0.449	0.07515242
AFTPH	54812	71-pteVam1	1	0.4183	0.34183525
AFTPH	54812	95-ornAna1	1	0.3342	0.28408941
AFTPH	54812	chiLan1-58	1	0.839	0.56785755
AFTPH	54812	chiLan1-pteVam1	1	0.3527	0.297212
AFTPH	54812	hetGla2-pteAle1	1	0.2637	0.23179603
AFTPH	54812	odoRosDiv1-58	1	0.4409	0.35654295
AFTPH	54812	odoRosDiv1-hetGla2	3	1.7981	0.26887018
AFTPH	54812	orcOrc1-hetGla2	1	0.2074	0.18730549
AHSA2	130827	chiLan1-dasNov3	1	0.7681	0.53610637
AHSA2	130827	lepWed1-octDeg1	1	0.3414	0.28922546
AHSA2	130827	loxAfr3-ornAna1	1	0.9033	0.59476981
AHSA2	130827	odoRosDiv1-conCri1	1	0.8869	0.58806924
AHSA2	130827	odoRosDiv1-pteAle1	1	0.0402	0.0394027
AHSA2	130827	pteAle1-conCri1	1	0.3514	0.29629778
AHSA2	130827	turTru2-76	1	0.4219	0.34420038
AIMP2	7965	66-84	1	0.297	0.25695599
AIMP2	7965	loxAfr3-ornAna1	2	1.7263	0.69281946
ALDOA	226	97-94	1	0.4701	0.37506023

ALDOA	226	97-loxAfr3	1	0.9007	0.59371484
ALDOA	226	lepWed1-chiLan1	2	0.6694	0.145241
ALDOA	226	pteAle1-sorAra2	1	0.3838	0.31873233
AMER1	139285	hetGla2-pteVam1	1	0.6079	0.4555069
AMER1	139285	lepWed1-71	1	0.689	0.4979221
AMER1	139285	odoRosDiv1-58	2	0.9915	0.26111418
AMER1	139285	pteVam1-58	1	0.4527	0.36409112
AMER1	139285	pteVam1-83	1	0.949	0.61287204
AMHR2	269	66-71	1	0.8415	0.56893656
AMHR2	269	66-octDeg1	1	0.9222	0.60235674
AMHR2	269	97-94	1	0.9724	0.62182567
AMHR2	269	chiLan1-71	2	1.9843	0.72720476
AMHR2	269	hetGla2-pteVam1	1	0.4392	0.35544814
AMHR2	269	lepWed1-57	1	0.7059	0.50633592
AMHR2	269	lepWed1-71	1	0.4459	0.35975221
AMHR2	269	lepWed1-76	1	0.8066	0.55362684
AMHR2	269	lepWed1-chiLan1	1	0.3982	0.32847229
AMHR2	269	octDeg1-pteVam1	1	0.3503	0.29552329
AMHR2	269	odoRosDiv1-dasNov3	3	2.8579	0.7656344
AMHR2	269	pteVam1-57	1	0.4878	0.38602434
AMHR2	269	pteVam1-dasNov3	1	0.8752	0.58322134
ANKRD11	29123	66-84	2	1.5553	0.67163568
ANKRD11	29123	66-orcOrc1	1	0.2275	0.20347758
ANKRD11	29123	odoRosDiv1-84	4	1.8896	0.12352494
ANKRD11	29123	orcOrc1-eriEur2	2	1.2651	0.3607637
AP3B2	8120	orcOrc1-83	1	0.6029	0.45277761
AR	367	lepWed1-71	1	0.7801	0.54163983
AR	367	odoRosDiv1-58	1	0.4979	0.39219429
AR	367	odoRosDiv1-hetGla2	2	1.1126	0.30558158
AR	367	odoRosDiv1-octDeg1	1	0.913	0.59868154
ARHGAP32	9743	orcOrc1-76	1	0.8042	0.55255426
ARID5B	84159	lepWed1-84	1	0.7656	0.53494519

ARID5B	84159	turTru2-83	1	0.69	0.49842393
ARID5B	84159	turTru2-chiLan1	1	0.3999	0.32961292
ATE1	11101	lepWed1-chiLan1	1	0.2925	0.25360475
ATE1	11101	odoRosDiv1-orcOrc1	1	0.0699	0.06751294
ATE1	11101	orcOrc1-dasNov3	1	0.3918	0.32416073
ATE1	11101	pteAle1-57	1	0.8611	0.57730314
ATE1	11101	pteAle1-83	1	0.3735	0.31167901
ATE1	11101	turTru2-sorAra2	3	1.6699	0.23485296
ATRX	546	84-57	2	1.3996	0.40802918
ATRX	546	lepWed1-83	2	1.8372	0.7074025
ATRX	546	odoRosDiv1-58	1	0.5545	0.42564063
ATRX	546	pteVam1-57	1	0.432	0.35079062
B3GNT2	10678	71-58	1	0.4823	0.38263817
B3GNT2	10678	lepWed1-hetGla2	1	0.6949	0.50087564
BAG3	9531	97-94	1	0.964	0.61863563
BAG3	9531	hetGla2-pteAle1	1	0.6458	0.47575702
BAG3	9531	lepWed1-57	1	0.9827	0.62570087
BAG3	9531	lepWed1-83	1	0.7966	0.54914072
BAG3	9531	odoRosDiv1-pteAle1	1	0.2924	0.25353011
BAG3	9531	orcOrc1-dasNov3	1	0.4194	0.34255883
BAG3	9531	orcOrc1-eriEur2	1	0.5106	0.39986461
BAG3	9531	turTru2-84	1	0.2055	0.18575991
BCL11A	53335	echTel2-eleEdw1	3	2.6024	0.74909452
BIN3	55909	96-loxAfr3	1	0.2468	0.21870306
BIN3	55909	oryAfe1-94	1	0.4427	0.35770013
BMP4	652	oryAfe1-94	1	0.6796	0.49318032
BMP5	653	66-dasNov3	1	0.7685	0.53629189
C10orf90	118611	66-83	2	1.9287	0.7196885
C10orf90	118611	hetGla2-pteAle1	2	1.1482	0.31857543
C10orf90	118611	hetGla2-pteVam1	1	0.1226	0.11538256
C10orf90	118611	octDeg1-pteAle1	2	1.2803	0.36618172
C10orf90	118611	turTru2-57	1	0.8861	0.58773956

C10orf90	118611	turTru2-conCri1	1	0.6755	0.49109809
CAP2	10486	97-94	1	0.3883	0.32179115
CAP2	10486	pteVam1-conCri1	1	0.4812	0.3819587
CAPN3	825	97-94	2	1.0563	0.28494209
CAPN3	825	97-triMan1	2	1.9967	0.72888283
CAPN3	825	pteVam1-57	1	0.0951	0.090718
CCDC73	493860	71-84	3	2.9165	0.76981654
CCDC73	493860	lepWed1-84	1	0.9493	0.61298816
CCDC73	493860	lepWed1-pteVam1	2	0.8368	0.20449256
CCDC73	493860	orcOrc1-hetGla2	1	0.6549	0.48050599
CDC6	990	odoRosDiv1-58	1	0.9523	0.61414746
CDC6	990	pteVam1-83	1	0.7182	0.5123708
CEP68	23177	hetGla2-57	2	1.8919	0.71472977
CEP68	23177	lepWed1-84	1	0.6813	0.49404118
CEP68	23177	orcOrc1-84	1	0.2534	0.22384264
CEP68	23177	orcOrc1-hetGla2	1	0.629	0.46687534
CEP68	23177	pteAle1-83	1	0.5142	0.40202121
CEP68	23177	pteAle1-dasNov3	1	0.8516	0.57326839
CHD7	55636	octDeg1-pteVam1	2	1.5129	0.66675311
CHRM3	1131	66-pteAle1	1	0.3042	0.26228669
CHRM3	1131	turTru2-83	1	0.4049	0.33295649
CHRM3	1131	turTru2-conCri1	2	1.3992	0.40789107
CLDN11	5010	66-71	1	0.6387	0.47202165
CLDN11	5010	chiLan1-71	1	0.9196	0.60132152
CLDN11	5010	hetGla2-57	1	0.6321	0.46852547
CLDN11	5010	odoRosDiv1-chiLan1	1	0.4088	0.33555289
COL14A1	7373	turTru2-octDeg1	1	0.6946	0.50072588
COL1A2	1278	71-pteVam1	1	0.7391	0.52245649
COL1A2	1278	96-triMan1	1	0.9132	0.59876179
COL1A2	1278	chiLan1-pteVam1	1	0.4849	0.38424123
COL1A2	1278	lepWed1-84	1	0.9105	0.59767699
COL1A2	1278	orcOrc1-83	1	0.3666	0.30691318

COL1A2	1278	orcOrc1-conCri1	1	0.5437	0.41940393
COL1A2	1278	orcOrc1-dasNov3	1	0.592	0.44678026
COL1A2	1278	orcOrc1-sorAra2	1	0.5634	0.43072975
COL1A2	1278	pteAle1-sorAra2	1	0.6322	0.46857861
COL1A2	1278	turTru2-76	1	0.2832	0.2466309
COL2A1	1280	84-pteVam1	1	0.365	0.30580335
COL2A1	1280	orcOrc1-hetGla2	1	0.6168	0.46033138
COL2A1	1280	turTru2-84	1	0.4308	0.3500111
COLEC10	10584	97-94	1	0.1555	0.14401292
COLEC10	10584	odoRosDiv1-58	1	0.1993	0.18069593
COMMD1	150684	96-loxAfr3	1	0.733	0.51953457
COMMD1	150684	96-triMan1	1	0.3659	0.30642785
COMMD1	150684	odoRosDiv1-83	1	0.6027	0.45266816
CSMD3	114788	66-orcOrc1	1	0.2556	0.22554831
CSMD3	114788	pteAle1-57	1	0.718	0.51227327
CSTF3	1479	97-ornAna1	1	0.4307	0.3499461
CYP19A1	1588	66-58	1	0.915	0.59948337
CYP19A1	1588	71-84	1	0.8067	0.55367148
CYP19A1	1588	95-ornAna1	1	0.7848	0.54378907
CYP19A1	1588	orcOrc1-58	1	0.3288	0.28021304
CYP19A1	1588	orcOrc1-76	1	0.8744	0.58288779
DAGLB	221955	lepWed1-chiLan1	1	0.7537	0.52937797
DAGLB	221955	octDeg1-pteVam1	1	0.603	0.45283233
DAGLB	221955	odoRosDiv1-83	1	0.8906	0.58959057
DEPDC7	91614	hetGla2-57	1	0.3847	0.3193452
DEPDC7	91614	octDeg1-57	1	0.466	0.37249272
DES	1674	odoRosDiv1-57	1	0.879	0.5848021
DLG1	1739	66-71	1	0.4871	0.38559441
DLG1	1739	lepWed1-chiLan1	1	0.4858	0.38479516
DLG1	1739	lepWed1-hetGla2	1	0.7698	0.53689432
DLG1	1739	lepWed1-octDeg1	1	0.9171	0.60032358
DSCC1	79075	66-orcOrc1	1	0.0715	0.06900372

DSCC1	79075	octDeg1-pteVam1	1	0.3105	0.26691968
DSCC1	79075	odoRosDiv1-conCri1	1	0.9854	0.62671012
DSTN	11034	oryAfe1-triMan1	1	0.0971	0.09253474
ECHDC3	79746	lepWed1-84	1	0.3259	0.27812263
ECHDC3	79746	pteVam1-sorAra2	1	0.2383	0.21203373
EHBP1	23301	97-loxAfr3	1	0.6262	0.4653805
EHBP1	23301	chiLan1-pteAle1	1	0.3531	0.29749306
EHBP1	23301	hetGla2-57	1	0.9312	0.60591947
EHBP1	23301	turTru2-83	1	0.4312	0.35027105
EHBP1	23301	turTru2-eriEur2	1	0.9684	0.62030994
EIF2S1	1965	orcOrc1-76	1	0.1715	0.15759973
EIF3H	8667	71-84	1	0.7342	0.52011078
EIF3H	8667	97-triMan1	1	0.3862	0.32036541
EIF3H	8667	orcOrc1-conCri1	1	0.1325	0.12409707
EIF4EBP1	1978	66-83	1	0.2135	0.19224784
EIF4EBP1	1978	66-eriEur2	1	0.2941	0.25479803
EMC2	9694	chiLan1-pteVam1	1	0.8344	0.56586512
EMC2	9694	lepWed1-76	1	0.311	0.26728612
EMC2	9694	lepWed1-eriEur2	1	0.6182	0.46108639
ENPP2	5168	chiLan1-57	1	0.5857	0.44328398
ENPP2	5168	lepWed1-turTru2	1	0.1233	0.11600158
ENPP2	5168	turTru2-hetGla2	1	0.5295	0.41110065
ESR1	2099	71-84	1	0.4035	0.33202197
ESR1	2099	84-conCri1	2	1.9042	0.71638512
ESR1	2099	chiLan1-57	1	0.843	0.56958267
ESR1	2099	turTru2-57	1	0.3352	0.28480497
FAM204A	63877	66-71	1	0.4794	0.38084523
FAM204A	63877	66-84	1	0.3684	0.30815961
FAM204A	63877	66-octDeg1	1	0.807	0.55380536
FAM204A	63877	71-58	1	0.6715	0.48905841
FAM204A	63877	71-84	1	0.3732	0.31147249
FAM204A	63877	71-hetGla2	1	0.5837	0.44216943

FAM204A	63877	84-58	1	0.568	0.43334238
FAM204A	63877	97-94	1	0.5078	0.39818188
FAM204A	63877	97-triMan1	1	0.8622	0.57776785
FAM204A	63877	chiLan1-57	1	0.2542	0.22446332
FAM204A	63877	chiLan1-71	1	0.423	0.34492137
FAM204A	63877	chiLan1-sorAra2	3	2.7504	0.75829915
FAM204A	63877	octDeg1-71	1	0.813	0.55647451
FAM204A	63877	pteAle1-eriEur2	1	0.1163	0.10979188
FAM45A	404636	66-eriEur2	1	0.9556	0.61541867
FAM45A	404636	84-58	1	0.5484	0.42212633
FAM45A	404636	pteVam1-eriEur2	1	0.515	0.40249941
FANCE	2178	66-84	1	0.922	0.6022772
FANCE	2178	76-57	1	0.6616	0.48397497
FANCE	2178	83-57	1	0.7396	0.5226952
FANCE	2178	hetGla2-pteVam1	1	0.8074	0.5539838
FANCE	2178	lepWed1-58	1	0.6359	0.47054124
FANCE	2178	lepWed1-orcOrc1	1	0.2881	0.25031338
FANCE	2178	odoRosDiv1-octDeg1	2	1.4042	0.4096164
FANCE	2178	turTru2-76	1	0.329	0.28035698
FAT1	2195	orcOrc1-58	1	0.4859	0.38485668
FAT1	2195	orcOrc1-chiLan1	2	1.8974	0.71546968
FBXL18	80028	pteAle1-sorAra2	1	0.3888	0.32213017
FGD1	2245	71-58	1	0.8694	0.580797
FGD1	2245	odoRosDiv1-76	1	0.6805	0.49363625
FGD1	2245	odoRosDiv1-dasNov3	1	0.9936	0.62975858
FGFR1	2260	66-71	1	0.9452	0.61139816
FHL3	2275	66-71	1	0.5694	0.43413514
FHL3	2275	83-58	1	0.4391	0.35538369
FHL3	2275	96-loxAfr3	1	0.1762	0.16154972
FHL3	2275	pteAle1-83	1	0.0346	0.03400826
FHL3	2275	pteAle1-sorAra2	1	0.1412	0.13168437
FOS	2353	84-57	1	0.2454	0.21760848

FOS	2353	97-loxAfr3	1	0.7803	0.54173149
FOXO1	2308	66-chiLan1	1	0.4358	0.35325294
FOXO1	2308	hetGla2-pteVam1	1	0.312	0.26801847
FOXP3	50943	66-octDeg1	1	0.7783	0.54081404
FOXP3	50943	hetGla2-58	1	0.8781	0.58442825
GFRA1	2674	66-chiLan1	1	0.4096	0.33608424
GFRA1	2674	71-58	1	0.6283	0.46650202
GFRA1	2674	lepWed1-58	1	0.2646	0.23248711
GFRA1	2674	lepWed1-pteAle1	1	0.1623	0.14981389
GFRA1	2674	pteAle1-58	1	0.1495	0.13886156
GLI1	2735	66-57	1	0.8926	0.59041057
GLI2	2736	66-57	2	1.6881	0.6879202
GLI2	2736	84-58	2	1.0801	0.29367798
GLI2	2736	odoRosDiv1-pteAle1	1	0.4534	0.3645361
GLI2	2736	turTru2-57	1	0.3494	0.29488897
GNRH1	2796	hetGla2-dasNov3	2	1.8807	0.7132246
GNRH1	2796	odoRosDiv1-octDeg1	1	0.1206	0.11361156
GNRHR	2798	66-octDeg1	1	0.6056	0.45425312
GNRHR	2798	odoRosDiv1-83	1	0.318	0.27239721
GNRHR	2798	odoRosDiv1-conCri1	1	0.9043	0.59517484
GNRHR	2798	odoRosDiv1-dasNov3	1	0.7465	0.52597727
GNRHR	2798	odoRosDiv1-pteAle1	1	0.0104	0.01034611
GNRHR	2798	pteAle1-83	1	0.0785	0.07549794
GNRHR	2798	pteAle1-conCri1	1	0.2217	0.19884433
GNRHR	2798	pteAle1-dasNov3	1	0.1845	0.16848006
GNRHR	2798	pteAle1-eriEur2	1	0.272	0.23814574
GNRHR	2798	turTru2-76	1	0.2823	0.24595256
GPC3	2719	odoRosDiv1-octDeg1	1	0.764	0.5342005
GRID2IP	392862	71-84	2	1.8925	0.71481047
GRID2IP	392862	orcOrc1-57	1	0.2881	0.25031338
HIPK3	10114	orcOrc1-76	1	0.2461	0.21815596
HMGB2	3148	58-dasNov3	1	0.3373	0.2863053

HMGB2	3148	83-76	1	0.6394	0.47239111
HOXA10	3206	71-84	1	0.1287	0.1207623
HOXA10	3206	84-conCri1	1	0.2671	0.23440349
HOXA10	3206	lepWed1-conCri1	1	0.509	0.39890363
HOXA10	3206	lepWed1-octDeg1	1	0.2132	0.19200548
HOXA11	3207	oryAfe1-ornAna1	1	0.7261	0.5162079
HOXA13	3209	echTel2-loxAfr3	1	0.7727	0.53823538
HOXA3	3200	orcOrc1-76	1	0.2594	0.22848564
HOXA3	3200	turTru2-hetGla2	1	0.8341	0.56573486
HOXA4	3201	oryAfe1-94	1	0.8278	0.56299035
HOXA5	3202	71-eriEur2	1	0.5656	0.43198077
HOXA5	3202	sorAra2-conCri1	1	0.52	0.40547945
HOXA6	3203	lepWed1-83	1	0.0861	0.08249752
HOXA7	3204	83-76	1	0.7961	0.54891523
HOXA7	3204	83-conCri1	1	0.7771	0.54026268
HOXA7	3204	96-loxAfr3	1	0.2021	0.18298678
HOXA7	3204	chiLan1-eriEur2	1	0.6861	0.49646396
HOXA7	3204	conCri1-dasNov3	1	0.8385	0.56764142
HOXA7	3204	odoRosDiv1-conCri1	1	0.5573	0.42724659
HOXA7	3204	orcOrc1-conCri1	1	0.1424	0.13272572
HOXA7	3204	orcOrc1-dasNov3	1	0.1027	0.09760235
HOXA7	3204	oryAfe1-loxAfr3	2	1.347	0.38973506
HOXA7	3204	turTru2-83	1	0.1276	0.11979461
HOXA7	3204	turTru2-conCri1	1	0.1899	0.17295817
HOXA7	3204	turTru2-eriEur2	1	0.431	0.35014109
HOXA9	3205	84-eriEur2	1	0.8057	0.55322493
HOXA9	3205	84-pteVam1	1	0.0628	0.06086872
HOXA9	3205	pteVam1-eriEur2	1	0.3848	0.31941326
HOXD1	3231	66-conCri1	1	0.7091	0.50791312
HOXD1	3231	66-octDeg1	1	0.6847	0.49575852
HOXD1	3231	octDeg1-57	1	0.7552	0.53008338
HOXD10	3236	71-sorAra2	1	0.7639	0.53415392

HOXD10	3236	76-conCri1	1	0.6245	0.46447087
HOXD12	3238	lepWed1-76	1	0.4153	0.33985779
HOXD12	3238	odoRosDiv1-dasNov3	1	0.7261	0.5162079
HOXD4	3233	84-dasNov3	1	0.7871	0.54483714
HOXD4	3233	odoRosDiv1-71	1	0.2894	0.25128734
HOXD4	3233	triMan1-ornAna1	1	0.6637	0.48505748
HOXD8	3234	71-76	1	0.3083	0.26530512
HOXD8	3234	83-sorAra2	1	0.6505	0.47821518
HOXD8	3234	chiLan1-eriEur2	1	0.3072	0.26449651
HOXD8	3234	hetGla2-sorAra2	1	0.894	0.59098359
HOXD9	3235	71-pteAle1	1	0.8254	0.56194027
HOXD9	3235	chiLan1-57	1	0.107	0.10147433
ID2	3398	orcOrc1-76	1	0.1304	0.12225574
ID2	3398	orcOrc1-83	1	0.2021	0.18298678
IGF1	3479	58-dasNov3	1	0.6261	0.46532704
IGF1	3479	chiLan1-58	1	0.3723	0.31085253
IGF1	3479	lepWed1-58	1	0.2256	0.20196275
IGF1	3479	lepWed1-dasNov3	1	0.4089	0.33561933
IGF1	3479	oryAfe1-triMan1	1	0.7107	0.50869983
IGFBP5	3488	chiLan1-76	1	0.5294	0.41104176
INPP5F	22876	odoRosDiv1-84	1	0.5234	0.40749739
IRAK1	3654	lepWed1-57	1	0.8129	0.55643015
IRAK1	3654	lepWed1-58	1	0.5673	0.43294558
IRAK1	3654	lepWed1-76	1	0.8896	0.58917995
IRAK1	3654	odoRosDiv1-octDeg1	1	0.9205	0.60168017
IRAK1	3654	pteAle1-dasNov3	1	0.3305	0.28143564
JAG1	182	chiLan1-58	1	0.7166	0.51158997
KCNV1	27012	66-76	1	0.7191	0.51280947
KCNV1	27012	97-ornAna1	1	0.3848	0.31941326
KCNV1	27012	odoRosDiv1-pteAle1	1	0.1521	0.14109761
KITLG	4254	66-conCri1	1	0.9118	0.59819967
KITLG	4254	76-58	1	0.5346	0.4140964

KITLG	4254	97-triMan1	1	0.5295	0.41110065
KITLG	4254	hetGla2-57	1	0.9367	0.60808096
KITLG	4254	lepWed1-dasNov3	2	1.2967	0.37200727
LBR	3930	84-pteVam1	1	0.1379	0.12881419
LBR	3930	96-triMan1	1	0.8288	0.56342714
LBR	3930	97-94	1	0.7886	0.54551938
LBR	3930	odoRosDiv1-71	2	1.4481	0.65967276
LBR	3930	turTru2-eriEur2	1	0.613	0.45827674
LGALS1	3956	66-83	1	0.405	0.33302319
LGALS1	3956	66-eriEur2	1	0.6561	0.48112901
LGALS1	3956	71-76	1	0.8575	0.57577869
LGALS1	3956	84-57	1	0.7475	0.52645105
LGALS1	3956	84-sorAra2	2	1.2478	0.35457615
LGALS1	3956	97-94	1	0.5211	0.40613307
LGALS1	3956	chiLan1-hetGla2	1	0.6728	0.4897222
LGALS1	3956	hetGla2-58	1	0.2774	0.24224866
LGALS1	3956	lepWed1-sorAra2	1	0.8942	0.59106538
LGALS1	3956	orcOrc1-58	1	0.095	0.09062707
LGALS1	3956	orcOrc1-hetGla2	1	0.1623	0.14981389
LGALS1	3956	oryAfe1-ornAna1	1	0.7111	0.50889631
LGALSL	29094	octDeg1-conCri1	1	0.8745	0.5829295
LIMS1	3987	hetGla2-58	1	0.3421	0.28972283
LIMS1	3987	lepWed1-dasNov3	1	0.4499	0.36230808
LIMS1	3987	lepWed1-sorAra2	1	0.909	0.59707305
LIMS1	3987	odoRosDiv1-76	1	0.9229	0.60263499
LMNA	4000	odoRosDiv1-84	1	0.5733	0.43633772
LMNA	4000	pteVam1-57	1	0.3017	0.2604401
LRP2	4036	lepWed1-pteVam1	1	0.7852	0.54397151
LRP2	4036	orcOrc1-octDeg1	3	1.8644	0.44166797
MAL2	114569	66-conCri1	1	0.6326	0.46879114
MAL2	114569	66-sorAra2	1	0.3974	0.32793485
MAL2	114569	71-76	2	1.939	0.72107926

MAL2	114569	76-58	1	0.8619	0.57764116
MAL2	114569	83-58	1	0.7207	0.51358835
MAL2	114569	hetGla2-57	1	0.8601	0.57688023
MAL2	114569	hetGla2-58	1	0.3958	0.32685869
MAL2	114569	lepWed1-sorAra2	1	0.7853	0.54401711
MAL2	114569	octDeg1-pteVam1	1	0.1514	0.14049617
MAP3K1	4214	66-84	1	0.9724	0.62182567
MAP3K1	4214	83-57	2	1.5826	0.67487025
MAP3K1	4214	97-94	1	0.8055	0.55313556
MBTPS2	51360	84-58	1	0.1972	0.17897359
MCMBP	79892	66-84	1	0.4083	0.33522058
MDH1	4190	66-octDeg1	1	0.9864	0.62708322
MDH1	4190	hetGla2-76	1	0.8591	0.5764569
MDH1	4190	pteVam1-sorAra2	1	0.5954	0.44865802
MECP2	4204	odoRosDiv1-octDeg1	1	0.403	0.3316879
MED30	90390	chiLan1-57	1	0.1674	0.1541388
MED30	90390	chiLan1-dasNov3	1	0.6452	0.47544238
MKX	283078	66-57	1	0.2274	0.20339792
MKX	283078	66-octDeg1	1	0.6981	0.50247029
MKX	283078	83-58	1	0.9534	0.61457166
MKX	283078	lepWed1-58	1	0.0979	0.09326043
MKX	283078	lepWed1-83	1	0.3417	0.28943866
MMP14	4323	84-58	1	0.8934	0.59073811
MMP14	4323	lepWed1-71	1	0.6105	0.45692074
MMP14	4323	lepWed1-76	1	0.5202	0.40559834
MMP9	4318	71-pteAle1	1	0.4279	0.3481234
MMP9	4318	96-triMan1	2	1.6778	0.68661305
MMP9	4318	lepWed1-71	2	0.885	0.22203378
MMP9	4318	lepWed1-chiLan1	1	0.649	0.47743192
MMP9	4318	odoRosDiv1-hetGla2	3	1.3791	0.16147439
MMP9	4318	orcOrc1-58	1	0.4056	0.33342326
MMP9	4318	pteAle1-57	1	0.4365	0.3537055

MMP9	4318	pteAle1-eriEur2	2	1.1638	0.32425133
MMP9	4318	turTru2-58	1	0.3103	0.26677304
MMP9	4318	turTru2-83	1	0.4408	0.3564786
MMP9	4318	turTru2-84	1	0.1608	0.14853765
MMP9	4318	turTru2-dasNov3	1	0.8838	0.58679027
MPP7	143098	66-57	1	0.5214	0.4063112
MPP7	143098	66-76	1	0.9336	0.60686413
MPP7	143098	97-ornAna1	2	1.5383	0.66965644
MPP7	143098	orcOrc1-84	1	0.0908	0.08679967
MPP7	143098	orcOrc1-conCri1	1	0.9226	0.60251576
MSX2	4488	76-57	1	0.4581	0.36751578
MYF6	4618	odoRosDiv1-sorAra2	1	0.4837	0.38350188
MYH7	4625	orcOrc1-octDeg1	1	0.5596	0.42856241
MYL3	4634	83-58	1	0.6556	0.48086951
MYL3	4634	84-dasNov3	2	1.6194	0.6793305
MYL3	4634	95-ornAna1	1	0.6834	0.49510258
MYL3	4634	96-94	1	0.3762	0.31353497
MYL3	4634	96-loxAfr3	1	0.5971	0.4495945
MYL3	4634	96-triMan1	1	0.7677	0.53592078
MYL3	4634	hetGla2-pteVam1	1	0.0863	0.08268101
MYL3	4634	pteAle1-conCri1	1	0.5987	0.45047444
MYOG	4656	97-94	1	0.091	0.08698229
NCAM1	4684	71-pteAle1	1	0.8378	0.56733867
NCAM1	4684	96-triMan1	1	0.9041	0.59509386
NCAM1	4684	turTru2-conCri1	1	0.7091	0.50791312
NOTCH2	4853	84-pteAle1	1	0.8741	0.58276264
NOV	4856	96-loxAfr3	1	0.3609	0.3029513
NOV	4856	97-triMan1	1	0.6516	0.47878883
NOV	4856	hetGla2-58	1	0.7127	0.50968145
NOV	4856	odoRosDiv1-71	1	0.7892	0.54579198
NR0B1	190	71-pteVam1	1	0.1188	0.11201462
NR0B1	190	lepWed1-57	2	1.0465	0.28134149

NR0B1	190	lepWed1-chiLan1	1	0.8532	0.57395061
NR0B1	190	odoRosDiv1-pteAle1	1	0.0928	0.08862424
NR0B1	190	orcOrc1-76	1	0.3631	0.30448312
NR0B1	190	orcOrc1-sorAra2	1	0.9505	0.6134523
NR1D2	9975	66-chiLan1	1	0.4208	0.34347861
NR1D2	9975	66-dasNov3	1	0.9648	0.6189406
NR1D2	9975	83-58	1	0.8668	0.57970566
NR1D2	9975	odoRosDiv1-84	1	0.0921	0.08798606
NR1D2	9975	pteAle1-sorAra2	1	0.3107	0.26706628
NR4A1	3164	96-94	1	0.243	0.21572849
NR4A1	3164	odoRosDiv1-58	1	0.5023	0.39486276
NR4A1	3164	oryAfe1-94	3	2.0007	0.45875363
NR5A1	2516	66-58	1	0.3809	0.31675379
NR5A1	2516	66-octDeg1	1	0.6931	0.49997641
NSD1	64324	96-triMan1	1	0.5142	0.40202121
NSD1	64324	lepWed1-84	1	0.9916	0.62901736
NSD1	64324	orcOrc1-chiLan1	1	0.1616	0.14921855
NSD1	64324	turTru2-83	1	0.6992	0.50301727
NSMCE4A	54780	hetGla2-57	2	1.6802	0.68691707
NSMCE4A	54780	octDeg1-pteAle1	1	0.2277	0.20363687
NUDCD1	84955	odoRosDiv1-58	1	0.8216	0.56027247
NUDCD1	84955	pteVam1-58	1	0.1227	0.11547102
OCRL	4952	96-triMan1	1	0.4806	0.38158777
OCRL	4952	octDeg1-pteVam1	1	0.4442	0.35866286
OCRL	4952	odoRosDiv1-71	1	0.9705	0.62110646
OCRL	4952	orcOrc1-76	1	0.1793	0.16414489
OCRL	4952	orcOrc1-octDeg1	1	0.137	0.12802977
ORC1	4998	66-58	1	0.7137	0.51017152
ORC1	4998	96-loxAfr3	3	1.7377	0.25276031
ORC1	4998	lepWed1-58	1	0.7778	0.54058439
ORC1	4998	lepWed1-71	2	1.7233	0.69243196
ORC1	4998	lepWed1-chiLan1	2	1.3981	0.40751116

ORC1	4998	odoRosDiv1-58	1	0.9866	0.6271578
ORC1	4998	odoRosDiv1-orcOrc1	1	0.2174	0.19539194
ORC1	4998	orcOrc1-76	1	0.9068	0.59618564
ORC6	23594	66-76	1	0.5877	0.4443963
ORC6	23594	71-58	1	0.2018	0.18274164
ORC6	23594	71-pteVam1	1	0.1977	0.179384
ORC6	23594	84-sorAra2	2	1.1773	0.32915316
ORC6	23594	chiLan1-57	1	0.6117	0.45757204
ORC6	23594	lepWed1-83	1	0.6014	0.45195616
ORC6	23594	odoRosDiv1-84	1	0.1232	0.11591317
ORC6	23594	pteVam1-sorAra2	1	0.9679	0.62012005
OTX1	5013	lepWed1-octDeg1	1	0.5154	0.40273836
PAPOLG	64895	hetGla2-58	1	0.9857	0.62682209
PARL	55486	chiLan1-71	1	0.984	0.62618715
PFN1	5216	83-57	1	0.1896	0.17271002
PFN1	5216	oryAfe1-triMan1	1	0.6989	0.50286815
PFN1	5216	triMan1-95	1	0.5843	0.44250403
PLPP4	196051	84-conCri1	1	0.3273	0.27913255
PLPP4	196051	97-ornAna1	1	0.4481	0.3611592
PLPP4	196051	97-triMan1	1	0.5932	0.44744373
PLPP4	196051	triMan1-ornAna1	1	0.7574	0.53111606
PMS2	5395	66-orcOrc1	1	0.1304	0.12225574
PMS2	5395	chiLan1-pteAle1	1	0.4005	0.33001503
PMS2	5395	lepWed1-turTru2	1	0.0715	0.06900372
PMS2	5395	odoRosDiv1-84	1	0.6863	0.49656466
PMS2	5395	orcOrc1-sorAra2	1	0.9663	0.61951176
PRDX3	10935	71-83	2	1.0894	0.29708764
PRDX3	10935	84-76	1	0.4695	0.37468515
PRDX3	10935	odoRosDiv1-sorAra2	1	0.7825	0.54273857
PREB	10113	84-76	1	0.806	0.55335894
PRLHR	2834	66-71	1	0.2611	0.2297961
PRLHR	2834	66-conCri1	1	0.4702	0.37512272

PRLHR	2834	66-eriEur2	1	0.6302	0.46751471
PRLHR	2834	66-octDeg1	1	0.2525	0.22314379
PRLHR	2834	84-58	1	0.9943	0.63001766
PRLHR	2834	hetGla2-58	3	2.4474	0.52914321
PROK2	60675	97-94	1	0.0666	0.06443065
PROSER2	254427	66-conCri1	2	1.8021	0.70273978
PROSER2	254427	hetGla2-pteVam1	1	0.1633	0.15066365
PROSER2	254427	lepWed1-hetGla2	1	0.9538	0.6147258
PROSER2	254427	orcOrc1-84	1	0.0307	0.03023354
PROSER2	254427	orcOrc1-dasNov3	1	0.3593	0.30183513
PROSER2	254427	turTru2-hetGla2	1	0.3245	0.27711129
PRRG4	79056	84-pteAle1	1	0.0803	0.07716055
PRRG4	79056	97-ornAna1	1	0.5823	0.44138792
PRRG4	79056	hetGla2-76	3	2.069	0.4683046
PRRG4	79056	lepWed1-eriEur2	2	1.6321	0.68089417
PRRG4	79056	lepWed1-hetGla2	1	0.6249	0.46468504
PTCH1	5727	turTru2-chiLan1	1	0.5446	0.41992623
PUS10	150962	orcOrc1-chiLan1	1	0.2846	0.24768488
QSER1	79832	66-chiLan1	2	1.8536	0.70959254
QSER1	79832	96-triMan1	1	0.1973	0.17905569
QSER1	79832	lepWed1-octDeg1	2	1.4582	0.66074295
QSER1	79832	pteAle1-conCri1	1	0.9235	0.60287334
RAB11FIP2	22841	71-58	1	0.3914	0.32389034
RAB11FIP2	22841	83-58	1	0.3877	0.3213841
RAB11FIP2	22841	eleEdw1-94	1	0.9518	0.61395448
RAB18	22931	oryAfe1-94	1	0.5702	0.43458766
RAB23	51715	76-57	1	0.1794	0.16422848
RAB23	51715	83-57	1	0.4761	0.37879864
RAB23	51715	turTru2-sorAra2	1	0.218	0.19587456
RAB29	8934	84-58	1	0.6242	0.46431019
RAB2B	84932	orcOrc1-conCri1	1	0.3103	0.26677304
RAB3GAP2	25782	octDeg1-pteVam1	1	0.5405	0.41754305

RAD21	5885	96-ornAna1	1	0.4498	0.36224431
RAD21	5885	97-94	1	0.7235	0.5149484
RAD21	5885	hetGla2-58	1	0.7609	0.53275428
RAD21	5885	odoRosDiv1-58	1	0.1491	0.13851704
RAD21	5885	odoRosDiv1-dasNov3	1	0.7025	0.50465461
RALBP1	10928	66-chiLan1	2	0.254	0.02728248
RALBP1	10928	66-octDeg1	1	0.575	0.43729513
RET	5979	orcOrc1-hetGla2	1	0.6304	0.46762119
RET	5979	pteAle1-83	1	0.8263	0.56233434
RET	5979	pteVam1-conCri1	1	0.9682	0.620234
RNF216	54476	71-84	1	0.8584	0.57616032
RNF216	54476	96-triMan1	1	0.3552	0.29896678
RNF216	54476	odoRosDiv1-turTru2	1	0.086	0.08240577
RNF216	54476	orcOrc1-76	1	0.3612	0.30316038
RNF216	54476	orcOrc1-conCri1	1	0.3684	0.30815961
RNF216	54476	pteAle1-dasNov3	1	0.6505	0.47821518
RPS6KB1	6198	71-dasNov3	1	0.8902	0.58942637
RXFP1	59350	66-chiLan1	1	0.8142	0.55700642
SAMD12	401474	71-84	1	0.4488	0.36160624
SAMD12	401474	83-58	1	0.2063	0.18641104
SAMD12	401474	96-triMan1	1	0.1551	0.14367046
SAMD12	401474	octDeg1-sorAra2	2	1.5419	0.67007324
SAMD12	401474	odoRosDiv1-57	1	0.1199	0.11299087
SAMD12	401474	odoRosDiv1-conCri1	1	0.6157	0.45973742
SEC23IP	11196	lepWed1-pteVam1	1	0.1492	0.13860318
SEMA3A	10371	71-57	1	0.5999	0.45113348
SEMA3A	10371	71-58	1	0.669	0.48777946
SEMA3A	10371	lepWed1-orcOrc1	1	0.0517	0.05038629
SERTAD2	9792	66-dasNov3	1	0.7786	0.54095177
SERTAD2	9792	lepWed1-58	1	0.0368	0.03613111
SERTAD2	9792	octDeg1-57	1	0.4913	0.3881695
SERTAD2	9792	pteVam1-sorAra2	1	0.5793	0.43970957

SLC30A8	169026	66-57	1	0.3052	0.26302404
SLC30A8	169026	84-76	2	1.4549	0.66039185
SLC30A8	169026	lepWed1-84	1	0.3768	0.31394673
SLC30A8	169026	lepWed1-hetGla2	2	1.3398	0.38721096
SLC30A8	169026	odoRosDiv1-58	1	0.3385	0.28716122
SLC9A4	389015	66-turTru2	1	0.2278	0.2037165
SLC9A4	389015	odoRosDiv1-57	1	0.8454	0.57061443
SOX2	6657	eleEdw1-triMan1	1	0.9514	0.61380004
SPRED2	200734	83-57	1	0.541	0.4178342
SPRED2	200734	lepWed1-eriEur2	2	1.2183	0.34397663
SPRED2	200734	turTru2-dasNov3	1	0.1819	0.1663153
SYBU	55638	chiLan1-57	2	1.5224	0.66783119
SYBU	55638	lepWed1-57	1	0.8327	0.56512646
SYBU	55638	lepWed1-84	1	0.5776	0.43875626
SYBU	55638	odoRosDiv1-58	1	0.6771	0.49191169
SYBU	55638	odoRosDiv1-71	1	0.9049	0.59541766
SYBU	55638	pteVam1-dasNov3	1	0.3835	0.31852792
SYBU	55638	turTru2-eriEur2	1	0.3197	0.27363309
SYCE3	644186	83-dasNov3	1	0.6474	0.47659514
SYCE3	644186	93-ornAna1	1	0.8651	0.57899055
SYCE3	644186	eleEdw1-loxAfr3	1	0.6933	0.5000764
SYCE3	644186	oryAfe1-loxAfr3	1	0.8076	0.55407299
TAF2	6873	hetGla2-57	1	0.4644	0.3714879
TAF3	83860	pteAle1-58	1	0.4562	0.36631291
TAF3	83860	pteAle1-dasNov3	1	0.444	0.35853458
TAGLN2	8407	lepWed1-eriEur2	1	0.9931	0.62957341
TAGLN2	8407	orcOrc1-58	1	0.2525	0.22314379
TCP11L1	55346	odoRosDiv1-octDeg1	1	0.8182	0.55877485
TCP11L1	55346	orcOrc1-83	1	0.5605	0.42907647
TGFBR3	7049	lepWed1-orcOrc1	1	0.2485	0.22003014
TGFBR3	7049	orcOrc1-58	1	0.4654	0.3721161
TGFBR3	7049	pteAle1-conCri1	1	0.9655	0.61920724

TGFBR3	7049	pteAle1-eriEur2	1	0.9759	0.62314697
THBS4	7060	lepWed1-orcOrc1	1	0.1636	0.15091841
THBS4	7060	odoRosDiv1-58	1	0.8491	0.57220022
THBS4	7060	turTru2-pteAle1	1	0.0991	0.09434786
TIMP1	7076	58-dasNov3	1	0.4836	0.38344022
TIMP1	7076	66-eriEur2	1	0.8907	0.58963161
TIMP1	7076	chiLan1-71	1	0.6759	0.49130161
TIMP1	7076	lepWed1-chiLan1	1	0.9219	0.60223743
TIMP1	7076	odoRosDiv1-76	1	0.821	0.56000856
TIMP1	7076	odoRosDiv1-dasNov3	1	0.7772	0.54030865
TMEM17	200728	58-dasNov3	1	0.7022	0.50450598
TMEM17	200728	97-94	1	0.2509	0.22189982
TMEM17	200728	97-ornAna1	1	0.2281	0.20395535
TMEM17	200728	97-oryAfe1	1	0.9118	0.59819967
TMEM17	200728	chrAsi1-ornAna1	1	0.9795	0.6245012
TMEM17	200728	lepWed1-conCri1	1	0.8805	0.58542443
TMEM17	200728	lepWed1-dasNov3	1	0.9036	0.59489136
TMEM17	200728	loxAfr3-ornAna1	1	0.625	0.46473857
TMEM17	200728	octDeg1-76	1	0.9804	0.624839
TMEM17	200728	octDeg1-pteVam1	1	0.136	0.12715737
TMEM17	200728	pteVam1-conCri1	1	0.3044	0.26243422
TMEM17	200728	pteVam1-eriEur2	1	0.3246	0.27718358
TMEM74	157753	84-58	1	0.2694	0.23616234
TMEM74	157753	96-ornAna1	1	0.7604	0.5325206
TMEM74	157753	chiLan1-57	1	0.6942	0.50052613
TMEM74	157753	chiLan1-58	1	0.7064	0.50658269
TMEM74	157753	odoRosDiv1-57	1	0.3827	0.31798253
TMEM74	157753	odoRosDiv1-chiLan1	1	0.8031	0.5520618
TNFRSF11B	4982	96-ornAna1	1	0.3848	0.31941326
TNFRSF11B	4982	orcOrc1-dasNov3	1	0.832	0.56482194
TPM1	7168	84-conCri1	1	0.3701	0.30933474
TPM1	7168	84-eriEur2	1	0.637	0.47112332

TRDN	10345	chiLan1-71	3	2.3912	0.51951022
TRDN	10345	lepWed1-58	1	0.2256	0.20196275
TRDN	10345	lepWed1-chiLan1	1	0.4456	0.3595601
TRDN	10345	lepWed1-hetGla2	1	0.6818	0.4942941
TRDN	10345	lepWed1-octDeg1	1	0.7503	0.52777514
TRDN	10345	lepWed1-sorAra2	4	1.7421	0.09958758
TRDN	10345	octDeg1-58	2	1.7294	0.69322033
TRDN	10345	orcOrc1-dasNov3	1	0.2779	0.24262745
TRDN	10345	orcOrc1-eriEur2	1	0.3138	0.26933485
TRDN	10345	pteVam1-57	1	0.2321	0.20713317
TRHR	7201	hetGla2-57	1	0.9549	0.61514937
TRPS1	7227	odoRosDiv1-chiLan1	1	0.9594	0.61687731
TRPS1	7227	turTru2-conCri1	1	0.5003	0.39365127
TRPS1	7227	turTru2-hetGla2	1	0.6877	0.49726898
USP6NL	9712	66-71	1	0.6521	0.47904937
USP6NL	9712	66-83	1	0.991	0.6287947
USP6NL	9712	lepWed1-83	2	1.1761	0.32871783
USP6NL	9712	odoRosDiv1-57	1	0.9587	0.61660903
USP6NL	9712	pteAle1-conCri1	1	0.777	0.54021671
USP6NL	9712	pteVam1-83	1	0.3643	0.30531724
USP6NL	9712	pteVam1-eriEur2	1	0.7432	0.52441041
USP6NL	9712	turTru2-hetGla2	1	0.2956	0.255915
UTP23	84294	66-conCri1	1	0.895	0.5913924
UTP23	84294	lepWed1-58	1	0.3221	0.27537428
VPS54	51542	66-83	1	0.6557	0.48092142
VPS54	51542	83-58	1	0.6245	0.46447087
VPS54	51542	turTru2-hetGla2	1	0.3031	0.26147476
VRK2	7444	66-57	1	0.7696	0.53680169
VRK2	7444	66-71	2	1.614	0.67866929
VRK2	7444	84-conCri1	4	3.1869	0.56748056
VRK2	7444	96-triMan1	1	0.6823	0.49454689
VRK2	7444	octDeg1-57	2	1.7447	0.69520546

VRK2	7444	pteVam1-eriEur2	1	0.9069	0.59622601
WDPCP	51057	66-58	1	0.3961	0.3270606
WDPCP	51057	66-chiLan1	1	0.5477	0.42172168
WDPCP	51057	chiLan1-58	1	0.9873	0.6274187
WDPCP	51057	lepWed1-turTru2	1	0.0322	0.0316871
WDPCP	51057	odoRosDiv1-76	2	1.869	0.71165467
WDPCP	51057	turTru2-chiLan1	1	0.1042	0.09895493
WDR11	55717	71-pteAle1	1	0.4596	0.36846379
WDR11	55717	hetGla2-pteAle1	1	0.5181	0.40434879
WIPF1	7456	odoRosDiv1-pteAle1	1	0.236	0.21021933
WIPF1	7456	pteAle1-eriEur2	1	0.8686	0.58046151
WIPI2	26100	66-76	1	0.7846	0.54369781
WIPI2	26100	84-eriEur2	1	0.8127	0.55634143
ZMPSTE24	10269	71-58	1	0.6196	0.46184034
ZMPSTE24	10269	chiLan1-58	2	0.4164	0.06600254
ZMPSTE24	10269	chiLan1-71	1	0.851	0.57301227
ZNF12	7559	71-sorAra2	2	1.3903	0.40481388
ZNF12	7559	96-loxAfr3	1	0.3171	0.27174207
ZNF12	7559	orcOrc1-hetGla2	95	70.8375	0.00614554
ZNF12	7559	pteAle1-83	1	0.0672	0.06499182
ZNF2	7549	96-triMan1	1	0.3127	0.26853068
ZNF2	7549	octDeg1-58	1	0.704	0.50539707
AARD	441376	odoRosDiv1-84	1	0.2599	0.22887131
AARD	441376	oryAfe1-triMan1	1	0.4013	0.3305508
AARD	441376	pteAle1-conCri1	1	0.498	0.39225507
ADAMTS16	170690	lepWed1-orcOrc1	1	0.2831	0.24655556
ADAMTS16	170690	orcOrc1-83	1	0.9557	0.61545713
AMH	268	lepWed1-71	1	0.9591	0.61676236
AMH	268	odoRosDiv1-chiLan1	1	0.8737	0.58259571
ANOS1	3730	66-84	1	0.5949	0.44838228
ANOS1	3730	71-pteVam1	1	0.531	0.41198334
ANOS1	3730	lepWed1-71	1	0.8319	0.56477842

ANOS1	3730	octDeg1-pteVam1	1	0.9384	0.60874666
ANOS1	3730	odoRosDiv1-71	1	0.9433	0.61065911
ANOS1	3730	turTru2-83	1	0.4421	0.35731464
ARHGDIB	397	71-57	1	0.3549	0.29875644
ARHGDIB	397	71-76	1	0.79	0.5461552
ARHGDIB	397	76-57	1	0.5254	0.40868121
ARHGDIB	397	turTru2-76	1	0.1636	0.15091841
ARSE	415	lepWed1-orcOrc1	1	0.1352	0.12645881
ARSE	415	pteVam1-dasNov3	1	0.7453	0.5254081
ARSE	415	turTru2-83	1	0.1637	0.15100332
AXIN1	8312	71-84	1	0.6478	0.47680446
BICD2	23299	lepWed1-57	1	0.6965	0.5016736
BICD2	23299	orcOrc1-eriEur2	1	0.7516	0.52838863
BICD2	23299	pteAle1-dasNov3	1	0.6276	0.46612844
BICD2	23299	turTru2-sorAra2	1	0.6107	0.45702934
BMP7	655	66-pteVam1	1	0.1859	0.16964337
BNC1	646	lepWed1-84	1	0.7078	0.50727299
BNC1	646	odoRosDiv1-turTru2	1	0.1326	0.12418465
BNC1	646	orcOrc1-dasNov3	3	2.5133	0.74417302
BNC1	646	orcOrc1-hetGla2	2	0.4153	0.06570073
BNC1	646	turTru2-76	1	0.6716	0.4891095
BRCC3	79184	71-57	1	0.8505	0.57279872
BTBD1	53339	66-dasNov3	1	0.5342	0.41386199
BTBD16	118663	96-ornAna1	1	0.8518	0.57335372
BTBD16	118663	lepWed1-76	3	2.1866	0.48598889
BTBD16	118663	lepWed1-84	1	0.3572	0.30036744
BTBD16	118663	lepWed1-eriEur2	3	1.9027	0.44617395
BTBD16	118663	orcOrc1-hetGla2	1	0.2446	0.21698232
C15orf40	123207	66-57	1	0.6354	0.47027644
C15orf40	123207	66-84	2	0.2816	0.0329361
C15orf40	123207	66-chiLan1	1	0.186	0.16972641
C15orf40	123207	84-57	1	0.6953	0.50107525

C15orf40	123207	eleEdw1-triMan1	2	1.5923	0.67603522
C15orf40	123207	lepWed1-76	1	0.6407	0.47307655
C15orf40	123207	lepWed1-83	1	0.9722	0.62175003
C15orf40	123207	lepWed1-pteAle1	1	0.0868	0.08313955
C15orf40	123207	pteAle1-83	1	0.2551	0.22516099
C15orf40	123207	pteAle1-eriEur2	1	0.5267	0.40944943
CALCA	796	echTel2-triMan1	3	2.9512	0.77234488
CALCA	796	hetGla2-pteVam1	1	0.5168	0.40357394
CALCA	796	lepWed1-chiLan1	2	1.0006	0.26446185
CALCA	796	lepWed1-pteAle1	2	1.744	0.69511441
CALCA	796	orcOrc1-conCri1	1	0.1119	0.10586633
CALCA	796	pteVam1-83	1	0.5526	0.42454831
CALCA	796	turTru2-dasNov3	1	0.4008	0.330216
CAPG	822	lepWed1-84	1	0.2963	0.25643567
CAPG	822	octDeg1-58	1	0.2325	0.20745025
CAPG	822	pteVam1-57	1	0.5241	0.407912
CCND1	595	odoRosDiv1-76	1	0.896	0.5918008
CDC42EP4	23580	71-pteAle1	2	0.2969	0.03624985
CDC42EP4	23580	odoRosDiv1-chiLan1	1	0.3438	0.29092927
CDC42EP4	23580	pteAle1-dasNov3	1	0.4927	0.38902547
CDC42EP4	23580	pteAle1-sorAra2	1	0.8595	0.57662628
CDT1	81620	66-58	1	0.4576	0.36719945
CDT1	81620	71-58	1	0.8266	0.56246562
CDT1	81620	hetGla2-58	1	0.8692	0.58071316
CDT1	81620	lepWed1-orcOrc1	1	0.0271	0.02673609
CDT1	81620	turTru2-octDeg1	1	0.2273	0.20331826
CFL1	1072	lepWed1-71	1	0.1766	0.16188504
CFL1	1072	lepWed1-dasNov3	1	0.4574	0.36707288
COL5A1	1289	turTru2-84	1	0.6427	0.47412935
CPEB1	64506	71-76	1	0.49	0.38737361
CPEB1	64506	71-83	3	1.4066	0.1680995
CPEB1	64506	71-84	1	0.3034	0.26169628

CPEB1	64506	76-dasNov3	1	0.8163	0.55793573
CPEB1	64506	83-dasNov3	3	2.6488	0.75185295
CPEB1	64506	84-76	1	0.1361	0.12724465
CPEB1	64506	84-dasNov3	1	0.564	0.43107121
CPEB1	64506	97-94	3	0.6571	0.02913107
CPEB1	64506	97-ornAna1	1	0.9933	0.62964749
CPEB1	64506	chiLan1-71	1	0.5479	0.42183732
CPEB1	64506	hetGla2-dasNov3	2	1.2305	0.3483673
CPEB1	64506	hetGla2-pteAle1	1	0.7932	0.54760519
CPEB1	64506	loxAfr3-ornAna1	1	0.7333	0.51967869
CPEB1	64506	octDeg1-pteAle1	2	1.3501	0.39082038
CPEB1	64506	odoRosDiv1-71	1	0.6008	0.45162724
CPEB1	64506	odoRosDiv1-83	3	0.7726	0.04356426
CPEB1	64506	odoRosDiv1-hetGla2	1	0.3646	0.30552561
CPEB1	64506	odoRosDiv1-octDeg1	1	0.6176	0.46076295
CPEB1	64506	odoRosDiv1-pteAle1	1	0.7069	0.50682934
CPEB1	64506	triMan1-ornAna1	1	0.6756	0.49114898
CRHR1	1394	66-58	1	0.4539	0.36485376
EBP	10682	57-dasNov3	2	1.9314	0.72005297
EBP	10682	71-57	1	0.5826	0.44155548
EBP	10682	chiLan1-pteAle1	1	0.0657	0.06358825
EBP	10682	hetGla2-76	1	0.9641	0.61867376
EBP	10682	hetGla2-conCri1	3	2.1041	0.47343099
EBP	10682	odoRosDiv1-71	1	0.1878	0.17121955
EBP	10682	pteAle1-eriEur2	1	0.4487	0.36154239
EIF4E	1977	66-dasNov3	1	0.1779	0.16297388
EIF4E	1977	echTel2-triMan1	1	0.6699	0.48824025
EZR	7430	lepWed1-octDeg1	1	0.5356	0.41468201
EZR	7430	turTru2-sorAra2	1	0.6748	0.49074174
FAM103A1	83640	71-83	1	0.2134	0.19216706
FAM103A1	83640	chiLan1-dasNov3	2	1.6473	0.68278085
FAM161A	84140	66-84	1	0.6413	0.47339261

FAM161A	84140	hetGla2-57	2	1.7579	0.69692655
FAM161A	84140	lepWed1-76	2	1.6186	0.67923241
FAM161A	84140	lepWed1-hetGla2	1	0.9261	0.60390453
FAM161A	84140	lepWed1-octDeg1	1	0.9409	0.60972357
FAM161A	84140	octDeg1-57	2	1.7427	0.69494536
FAM161A	84140	orcOrc1-57	1	0.4871	0.38559441
FAM161A	84140	orcOrc1-83	1	0.957	0.61595671
FAM161A	84140	orcOrc1-hetGla2	1	0.5458	0.4206219
FAM161A	84140	pteVam1-58	1	0.6999	0.50336504
FARP2	9855	66-84	1	0.7528	0.52895422
FARP2	9855	66-octDeg1	3	2.2898	0.50254123
FARP2	9855	71-pteAle1	1	0.2203	0.19772192
FARP2	9855	96-triMan1	1	0.97	0.62091696
FARP2	9855	pteAle1-dasNov3	1	0.7034	0.50510022
FARP2	9855	turTru2-58	1	0.3872	0.32104471
FGFR2	2263	odoRosDiv1-octDeg1	1	0.9994	0.63189976
FKBP4	2288	97-loxAfr3	2	1.6227	0.67973566
FKBP4	2288	lepWed1-chiLan1	1	0.4395	0.35564148
FSD2	123722	odoRosDiv1-84	1	0.6577	0.48195854
FSD2	123722	odoRosDiv1-pteVam1	1	0.1532	0.14204189
FSD2	123722	pteVam1-83	1	0.7542	0.52961323
HOMER2	9455	66-76	2	1.3974	0.40726934
HOMER2	9455	66-octDeg1	1	0.7882	0.54533755
HOMER2	9455	lepWed1-58	1	0.4622	0.37010365
HOXA1	3198	83-57	1	0.5513	0.42379974
HRAS	3265	71-57	1	0.983	0.62581315
IGFBP3	3486	83-conCri1	3	2.1476	0.47996876
IGFBP3	3486	chiLan1-76	1	0.9738	0.62235474
IGFBP3	3486	hetGla2-pteAle1	1	0.1336	0.12506003
IGFBP3	3486	oryAfe1-loxAfr3	4	2.4607	0.31943212
INHA	3623	lepWed1-76	1	0.7918	0.54697139
INHA	3623	pteAle1-sorAra2	1	0.5021	0.39474172

INHA	3623	pteVam1-eriEur2	1	0.352	0.29671988
INHA	3623	turTru2-octDeg1	1	0.3963	0.32719518
INSL3	3640	71-84	1	0.2844	0.2475344
INSL3	3640	76-57	1	0.9615	0.61768102
INSL3	3640	84-57	1	0.1435	0.1336792
INSL3	3640	84-58	1	0.3682	0.30802123
INSL3	3640	84-76	1	0.639	0.47218002
INSL3	3640	84-sorAra2	1	0.8168	0.55815671
INSL3	3640	96-triMan1	1	0.9274	0.60441912
INSL3	3640	97-94	1	0.1229	0.11564791
INSL3	3640	lepWed1-71	1	0.2806	0.24466959
INSL3	3640	lepWed1-83	1	0.9612	0.61756631
INSL3	3640	lepWed1-conCri1	2	0.8774	0.21925927
INSL3	3640	octDeg1-76	2	1.4841	0.6635452
INSL3	3640	odoRosDiv1-76	1	0.7169	0.51173647
INSL3	3640	odoRosDiv1-sorAra2	1	0.8474	0.57147234
INSL3	3640	pteAle1-conCri1	1	0.2612	0.22987312
KANSL1	284058	84-76	1	0.8067	0.55367148
KANSL1	284058	chiLan1-58	2	1.7592	0.69709645
KISS1R	84634	chiLan1-pteAle1	1	0.2283	0.20411454
KISS1R	84634	hetGla2-57	2	1.6036	0.67740219
KISS1R	84634	odoRosDiv1-hetGla2	1	0.7179	0.51222449
MAPT	4137	71-83	2	1.9572	0.72353889
MAPT	4137	odoRosDiv1-84	1	0.9454	0.61147587
MID1IP1	58526	71-conCri1	2	1.3596	0.39414094
MID1IP1	58526	chiLan1-71	1	0.5346	0.4140964
MID1IP1	58526	chiLan1-eriEur2	2	1.4641	0.66137409
MID1IP1	58526	lepWed1-76	1	0.5734	0.43639408
MID1IP1	58526	octDeg1-58	1	0.5096	0.39926417
MSX1	4487	lepWed1-76	1	0.0662	0.06405634
MSX1	4487	turTru2-76	1	0.303	0.2614009
NANOS1	340719	93-ornAna1	1	0.5745	0.43701371

NANOS1	340719	odoRosDiv1-sorAra2	1	0.3771	0.31415251
NANOS1	340719	triMan1-ornAna1	1	0.7915	0.54683546
NDN	4692	lepWed1-chiLan1	1	0.1358	0.12698278
NDN	4692	pteVam1-eriEur2	1	0.0675	0.06527228
NDN	4692	turTru2-octDeg1	1	0.7547	0.52984836
NFKB1	4790	96-loxAfr3	1	0.6694	0.4879843
NFKB1	4790	96-triMan1	1	0.6365	0.47085882
NFKB1	4790	lepWed1-84	1	0.3012	0.26007023
NFKB1	4790	lepWed1-hetGla2	1	0.9289	0.60501204
NFKB1	4790	orcOrc1-83	1	0.8069	0.55376073
NHLH2	4808	loxAfr3-ornAna1	1	0.6639	0.48516046
NHLH2	4808	octDeg1-dasNov3	1	0.9838	0.62611238
OCM	654231	odoRosDiv1-hetGla2	1	0.2853	0.24821132
OCM	654231	odoRosDiv1-sorAra2	1	0.545	0.42015822
OLFM1	10439	83-58	2	1.1143	0.30620324
PGLS	25796	84-sorAra2	1	0.5919	0.44672494
PGLS	25796	odoRosDiv1-pteVam1	1	0.0463	0.04524451
PPP1CA	5499	orcOrc1-eriEur2	1	0.3611	0.3030907
PPP1CA	5499	pteVam1-83	1	0.1526	0.14152696
PTGDS	5730	66-eriEur2	1	0.7969	0.54927595
PTGDS	5730	71-83	2	1.4236	0.65713247
PTGDS	5730	83-58	2	1.3822	0.4020066
PTGDS	5730	84-eriEur2	1	0.8295	0.56373263
PTGDS	5730	octDeg1-58	1	0.9894	0.6282003
PTGDS	5730	odoRosDiv1-conCri1	1	0.5502	0.42316557
PTGDS	5730	odoRosDiv1-octDeg1	1	0.2588	0.2280226
PXN	5829	66-57	1	0.9617	0.61775748
RAD51C	5889	71-58	1	0.4604	0.36896882
RAD51C	5889	hetGla2-58	1	0.4577	0.36726273
RAD51C	5889	odoRosDiv1-hetGla2	1	0.6209	0.46253949
RXFP2	122042	66-58	1	0.655	0.48055794
RXFP2	122042	66-octDeg1	1	0.8774	0.58413725

RXFP2	122042	octDeg1-pteVam1	1	0.2806	0.24466959
RXFP2	122042	odoRosDiv1-hetGla2	1	0.7971	0.54936609
RXFP2	122042	odoRosDiv1-octDeg1	1	0.7637	0.53406074
RXFP2	122042	orcOrc1-octDeg1	1	0.1518	0.1408399
SCG5	6447	odoRosDiv1-83	1	0.6106	0.45697504
SFXN4	119559	66-71	1	0.5139	0.40184179
SFXN4	119559	96-triMan1	1	0.6587	0.48247632
SFXN4	119559	odoRosDiv1-conCri1	1	0.9918	0.62909155
SFXN4	119559	orcOrc1-dasNov3	1	0.5304	0.41163043
SFXN4	119559	orcOrc1-eriEur2	2	0.7296	0.16615674
SFXN4	119559	pteAle1-eriEur2	1	0.3335	0.2835881
SH3GL3	6457	66-dasNov3	2	1.0497	0.28251738
SH3GL3	6457	66-orcOrc1	1	0.0631	0.06115042
SH3GL3	6457	orcOrc1-dasNov3	1	0.7992	0.55031143
SH3GL3	6457	turTru2-76	1	0.2936	0.25442534
SOS1	6654	97-triMan1	1	0.9022	0.59432381
SOX8	30812	chiLan1-71	2	0.8858	0.222326
SPATA12	353324	66-71	1	0.4775	0.37966771
SPATA12	353324	lepWed1-hetGla2	1	0.7605	0.53256735
SPPL2C	162540	odoRosDiv1-orcOrc1	2	1.7329	0.69367348
STS	412	66-71	2	1.3269	0.38267712
STS	412	66-octDeg1	2	1.7749	0.69915374
STS	412	66-pteVam1	1	0.378	0.3147695
STS	412	71-pteVam1	1	0.979	0.6243134
STS	412	84-pteVam1	1	0.3964	0.32726245
STS	412	chiLan1-pteVam1	1	0.9625	0.61806315
STS	412	lepWed1-octDeg1	2	1.7907	0.70123363
STS	412	octDeg1-pteAle1	1	0.3974	0.32793485
SYNJ2BP	55333	71-conCri1	2	1.8178	0.70482091
SYNJ2BP	55333	83-sorAra2	1	0.9428	0.61046439
SYNJ2BP	55333	hetGla2-57	1	0.5735	0.43645044
SYNJ2BP	55333	lepWed1-71	1	0.4413	0.35680028

SYNJ2BP	55333	loxAfr3-ornAna1	1	0.7971	0.54936609
SYNJ2BP	55333	odoRosDiv1-sorAra2	1	0.9418	0.61007466
TACC2	10579	66-pteAle1	2	1.5007	0.66538288
TACC2	10579	turTru2-84	1	0.912	0.59828002
TACC2	10579	turTru2-chiLan1	2	1.957	0.72351185
TBCB	1155	71-57	1	0.7887	0.54556482
TBCB	1155	71-76	1	0.6499	0.47790202
TBCB	1155	76-57	2	1.3709	0.39807978
TBCB	1155	83-58	1	0.6797	0.493231
TBCB	1155	chiLan1-57	2	1.184	0.33158226
TBCB	1155	chiLan1-71	1	0.548	0.42189514
TBCB	1155	chiLan1-eriEur2	3	2.7517	0.75838491
TBCB	1155	lepWed1-57	2	0.6587	0.14158348
TBCB	1155	lepWed1-71	1	0.318	0.27239721
TBCB	1155	lepWed1-76	1	0.5427	0.41882305
TBCB	1155	lepWed1-octDeg1	1	0.5396	0.4170186
TBCB	1155	odoRosDiv1-chiLan1	1	0.5798	0.43998964
TBCB	1155	turTru2-57	1	0.9472	0.61217458
TBCB	1155	turTru2-76	1	0.7804	0.54177731
TBCB	1155	turTru2-chiLan1	1	0.6977	0.50227124
TM6SF1	53346	71-hetGla2	1	0.8141	0.55696212
TM6SF1	53346	chiLan1-pteAle1	1	0.0766	0.07373972
TM6SF1	53346	hetGla2-pteVam1	1	0.1861	0.16980943
TM6SF1	53346	lepWed1-71	1	0.9027	0.5945266
TM6SF1	53346	pteVam1-eriEur2	1	0.6644	0.48541782
TNK2	10188	66-84	1	0.7798	0.5415023
TNK2	10188	chiLan1-pteAle1	1	0.6381	0.47170477
TNNT2	7139	lepWed1-hetGla2	1	0.5106	0.39986461
TNNT2	7139	orcOrc1-dasNov3	1	0.404	0.33235588
TPM4	7171	odoRosDiv1-hetGla2	1	0.1622	0.14972887
VAMP7	6845	chiLan1-57	1	0.1939	0.17625973
VAMP7	6845	orcOrc1-76	1	0.9601	0.6171454

VAMP7	6845	orcOrc1-conCri1	1	0.9035	0.59485085
WHAMM	123720	lepWed1-chiLan1	1	0.562	0.42993221
WHAMM	123720	octDeg1-pteAle1	1	0.5835	0.44205785
WHAMM	123720	odoRosDiv1-84	1	0.7214	0.51392872
WHAMM	123720	pteVam1-dasNov3	1	0.7885	0.54547393
WHAMM	123720	pteVam1-sorAra2	1	0.8738	0.58263745
WHAMM	123720	turTru2-83	1	0.1897	0.17279274
WHAMM	123720	turTru2-pteVam1	1	0.0111	0.01103862
WISP1	8840	76-57	1	0.9071	0.59630676
WISP1	8840	chiLan1-58	1	0.8253	0.56189646
WISP1	8840	odoRosDiv1-71	2	1.7402	0.69462048
ZNF214	7761	octDeg1-57	2	1.6669	0.68523671
ZNF214	7761	odoRosDiv1-hetGla2	2	1.3627	0.39522269
ZNF214	7761	orcOrc1-76	1	0.7427	0.52417255
ZNF215	7762	lepWed1-eriEur2	4	3.908	0.80024872
ZNF215	7762	odoRosDiv1-57	2	1.1888	0.33332096
ZNF215	7762	odoRosDiv1-hetGla2	1	0.9984	0.63153148
ZNF215	7762	odoRosDiv1-orcOrc1	1	0.1553	0.14384171
ZNF215	7762	orcOrc1-57	1	0.5502	0.42316557
ZNF215	7762	orcOrc1-83	1	0.705	0.50589143
ZNF215	7762	orcOrc1-hetGla2	1	0.4464	0.36007225

Table S11. Parallel and convergent amino acid substitutions in ascrotal UDT and IDT mammals.

Group	Protein	Branch pair	Obs substitution	Exp substitution	p value (possion test)
UDT	CPEB1	Internal node in Afrotheria (97) - Internal node in Afrotheria (94)	3	0.657	0.0291311
IDT	AHSA2	*Odobenus rosmarus divergens* - *Pteropus alecto*	1	0.040	0.0394027
	FHL3	*Pteropus alecto* - Internal node in Rodentia (83)	1	0.035	0.0340083
	GNRHR	*Odobenus rosmarus divergens* - *Pteropus alecto*	1	0.010	0.0103461
	PROSER2	*Orcinus orca* - Internal node in Rodentia (84)	1	0.031	0.0302335
	RALBP1	Internal node in pinniped (66) - *Chinchilla lanigera*	2	0.254	0.0272825
	SERTAD2	*Leptonychotes weddellii* - Internal node in Eulipotyphla (58)	1	0.037	0.0361311
	WDPCP	*Leptonychotes weddellii* - *Tursiops truncatus*	1	0.032	0.0316871
	C15orf40	Internal node in pinniped (66) - Internal node in Rodentia (84)	2	0.282	0.0329361
	CDC42EP4	Internal node in cetaceans (71) - *Pteropus alecto*	2	0.297	0.0362499
	CDT1	*Leptonychotes weddellii* - *Orcinus orca*	1	0.027	0.0267361
	CPEB1	*Odobenus rosmarus divergens* - Internal node in Rodentia (83)	3	0.773	0.0435643
	PGLS	*Odobenus rosmarus divergens* - *Pteropus vampyrus*	1	0.046	0.0452445
	WHAMM	*Tursiops truncates* - *Pteropus vampyrus*	1	0.011	0.0110386

Table S12. GO enrichment of genes exhibit parallel/convergent substitutions in IDT mammals.

ONTOLOGY	ID	Description	Gene Ratio	BgRatio	pvalue	p.adjust	qvalue	geneID	Count
MF	GO:0017049	GTP-Rho binding	2/12	17/17354	5.93E-05	0.00171883	0.00074868	23580/123720	2
MF	GO:0017048	Rho GTPase binding	3/12	161/17354	0.00016214	0.00235109	0.00102407	10928/23580/123720	3
MF	GO:0016500	protein-hormone receptor activity	1/12	12/17354	0.00826893	0.04975287	0.02167094	2798	1
MF	GO:0035925	mRNA 3'-UTR AU-rich region binding	1/12	12/17354	0.00826893	0.04975287	0.02167094	64506	1
MF	GO:0000900	translation repressor activity, nucleic acid binding	1/12	14/17354	0.00964097	0.04975287	0.02167094	64506	1
MF	GO:0017160	Ral GTPase binding	1/12	16/17354	0.01101127	0.04975287	0.02167094	10928	1
MF	GO:0005096	GTPase activator activity	2/12	284/17354	0.01580702	0.04975287	0.02167094	10928/23580	2
MF	GO:0090079	translation regulator activity, nucleic acid binding	1/12	24/17354	0.01647513	0.04975287	0.02167094	64506	1
MF	GO:0017091	AU-rich element binding	1/12	25/17354	0.01715616	0.04975287	0.02167094	64506	1
MF	GO:0030371	translation repressor activity	1/12	25/17354	0.01715616	0.04975287	0.02167094	64506	1
