# Supplementary material for: Rapid evolution and molecular convergence in cryptorchidism-related genes associated with inherently undescended testes in mammals
Source: BMC Ecol Evol. 2021 Feb 10;21:22. doi: 10.1186/s12862-021-01753-5 (PMC7877101; doi:10.1186/s12862-021-01753-5)
Supplement: Supplementary file 2 — Additional file 2: Fig S1. The expanded 62-mammal data set. Fig S2. Rapidly evolving genes in UDT mammals. Fig S3. Functional enrichment of genes that evolved significantly correlated to character of testis position (p value < 0.05). Fig S4. Top 10 GO function enrichment of positive selected genes in ascrotal mammals. Fig S5. The working tree with numbered internal nodes for identifying convergent/parallel amino acid substitutions. [file 12862_2021_1753_MOESM2_ESM.pdf]

Supplementary Figures

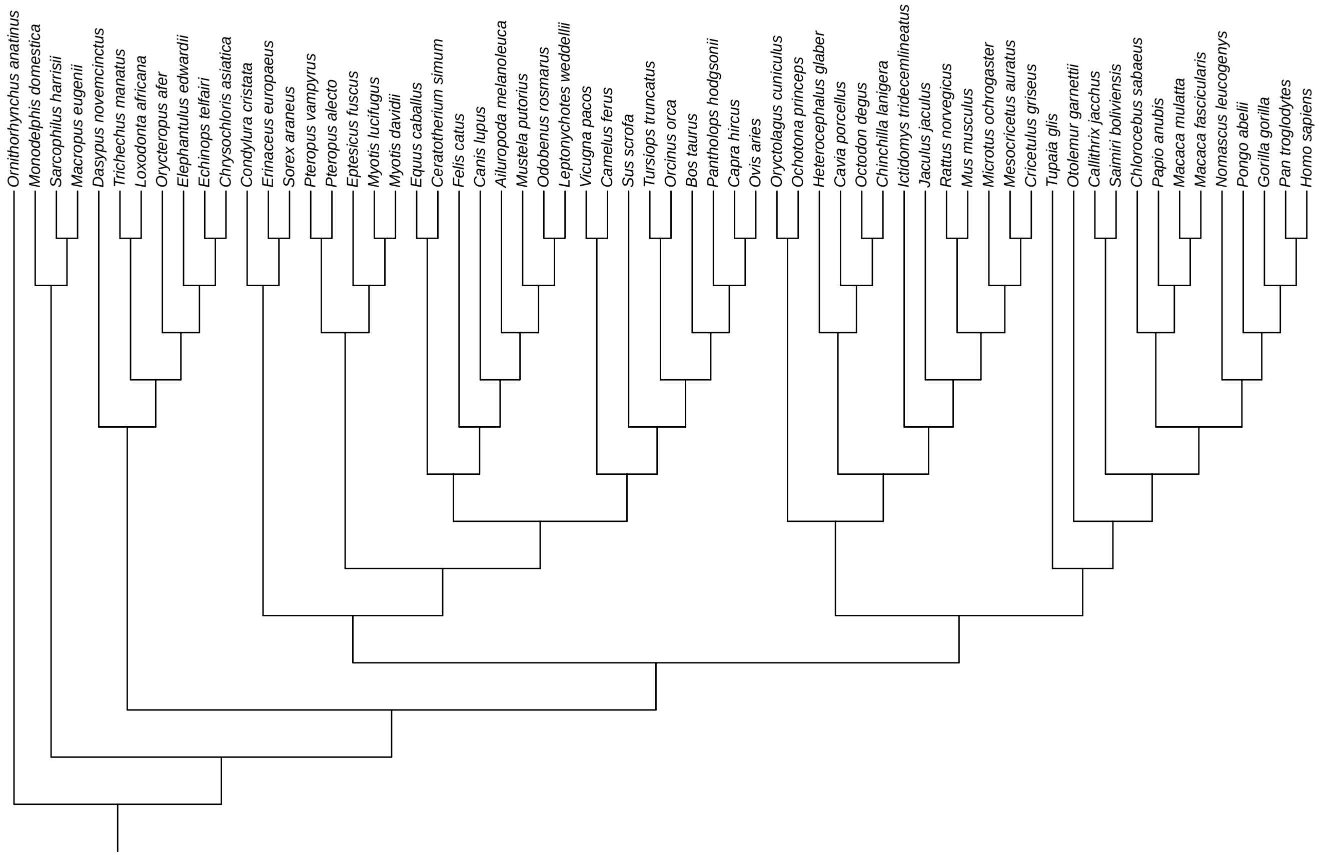

Figure S1. The expanded 62-mammal species tree.

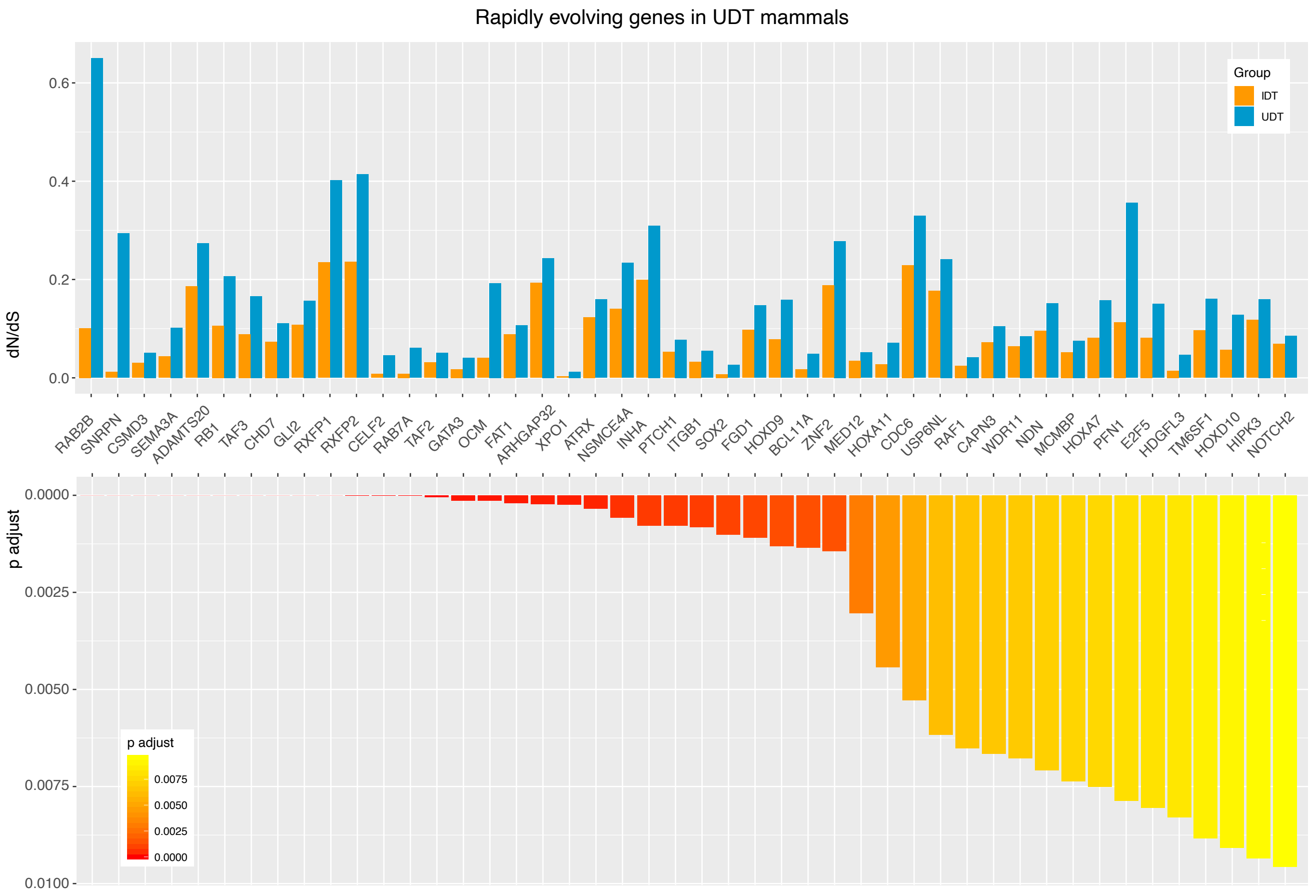

Figure S2. Rapidly evolving genes in UDT mammals. The upper half of this figure shows dN/dS ratios in UDT species and IDT species. In these 46 genes, dN/dS ratio in UDT species is higher than in IDT species. The bottom half of this figure demonstrates the p adjust (after fdr correction) values of 46 rapidly evolving genes.

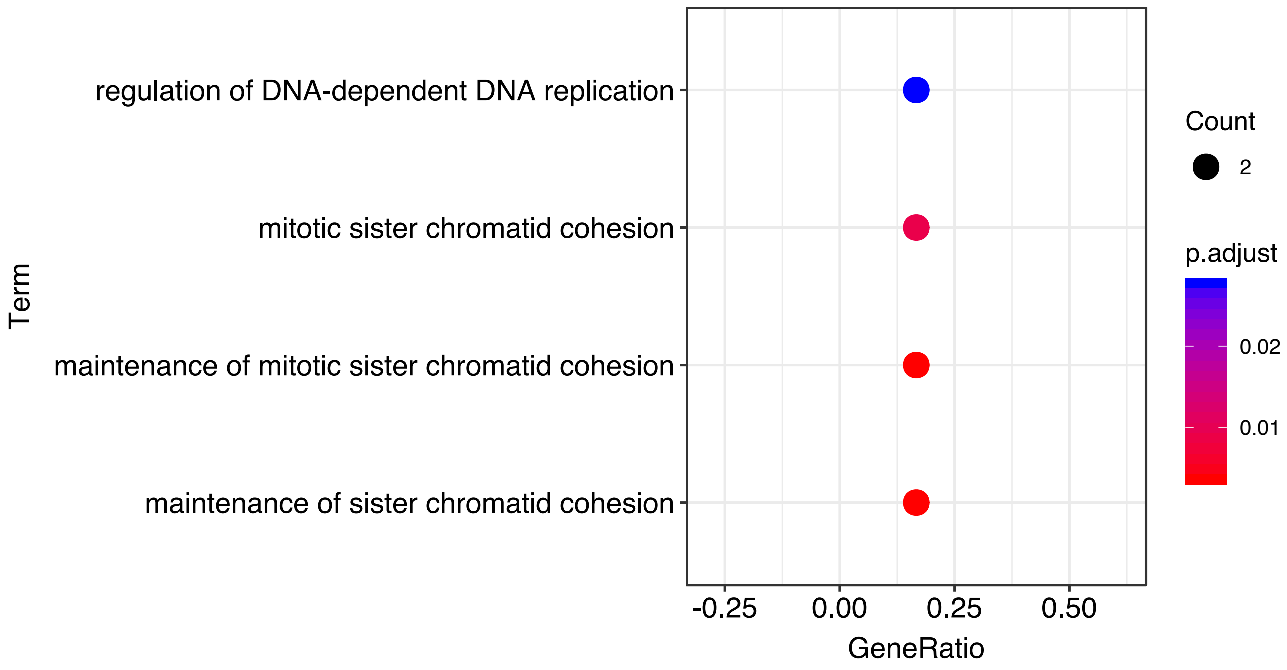

Figure S3. Functional enrichment of genes which evolved significantly correlated to character of testis position (p value < 0.05).

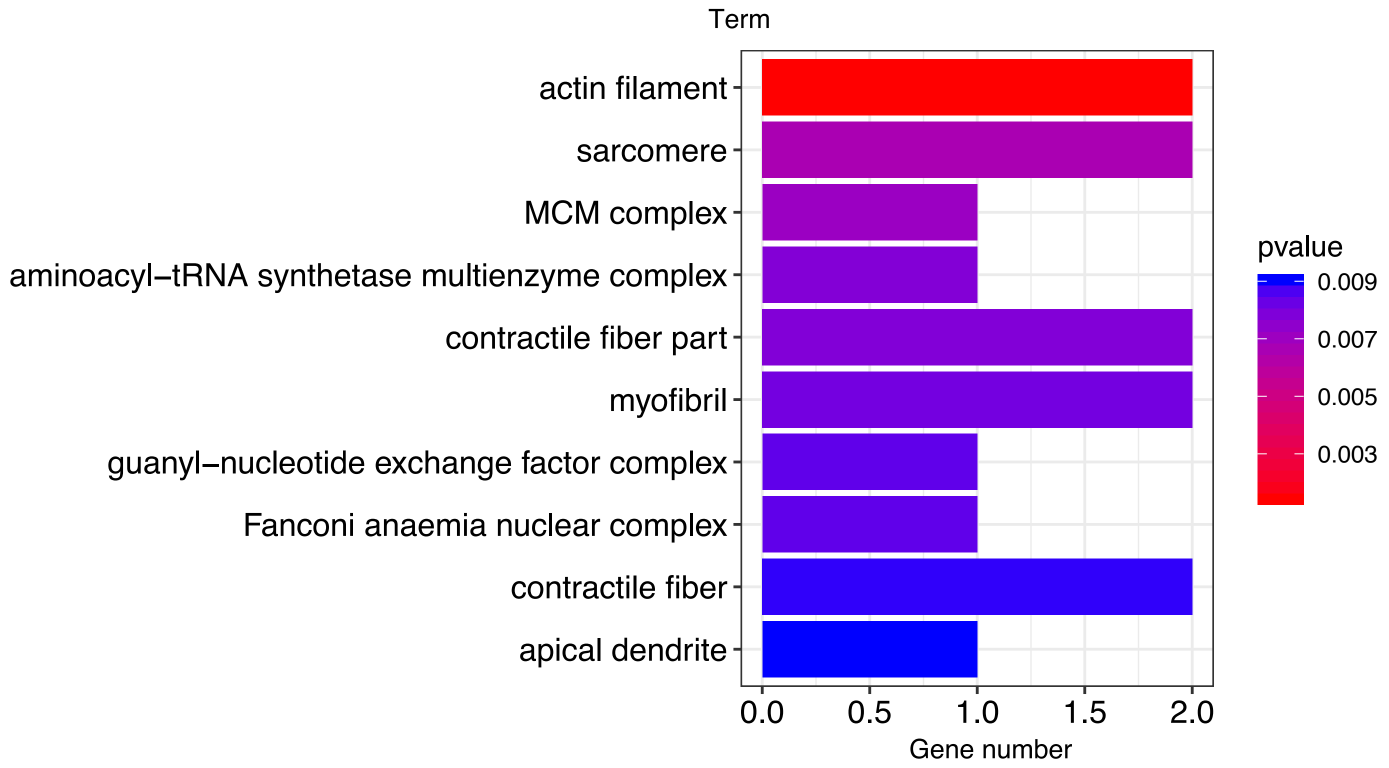

Figure S4. Top 10 GO function enrichment of positive selected genes in ascrotal IDT and UDT mammals. Enrichment terms are ordered by p values.

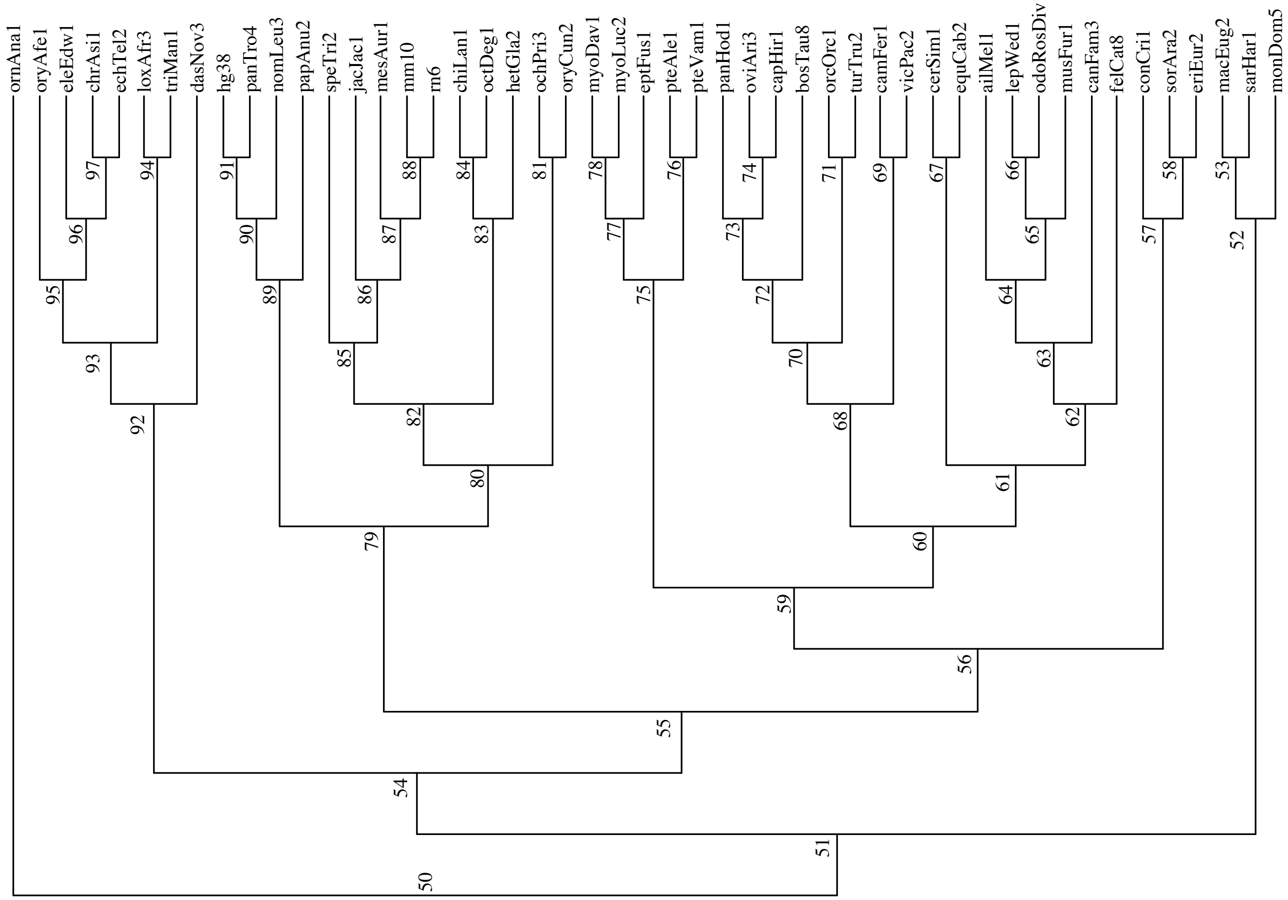

Figure S5. The working tree for identification of convergent/parallel amino acid substitutions. The internal nodes are numbered.
